# The Hepatoprotective Properties of the Revised Formulation of Dahuang Xiaoshi Tang, an Ancient Chinese Herbal Decoction, Are Probed by Integrated Metabolomics and Network Pharmacology

**DOI:** 10.3390/ph18101534

**Published:** 2025-10-13

**Authors:** Xiangpeng Kong, Xiaoyang Wang, Haiqin Ren, Yajun Yao, Hui Zhang, Huifeng Li, Huifang Li, Yangang Cheng, Zhuqing Song, Miaorong Pei, Karl Wah Keung Tsim

**Affiliations:** 1 College of Traditional Chinese Medicine and Food Engineering, Shanxi University of Chinese Medicine, Jinzhong 030619, China; kong_xiangpeng@sxtcm.edu.cn (X.K.); lihuifeng2046@163.com (H.L.); lihuifangzy@sxtcm.edu.cn (H.L.); chengyg1992@163.com (Y.C.); 19581931302@163.com (Z.S.); 2 Division of Life Science, Center for Chinese Medicine, The Hong Kong University of Science and Technology, Clear Water Bay, Hong Kong, China; xwangfr@connect.ust.hk; 3 College of Pharmacy, Harbin University of Commerce, Harbin 150076, China; haiqinren@163.com; 4 Department of Chinese Medicine, Shanxi Pharmaceutical Vocational College, Taiyuan 030031, China; yaoyajun0601@163.com; 5 School of Pharmacy, Faculty of Medicine, Macau University of Science and Technology, Macau SAR, China; 3240002660@student.must.edu.mo

**Keywords:** Dahuang Xiaoshi Tang, liver injury, metabolomics, network pharmacology, network toxicology, hepatoprotection

## Abstract

**Background**: Dahuang Xiaoshi Tang (DXT), an ancient Chinese herbal remedy dating back to 220 AD, as documented initially in “Treatise on Febrile and Miscellaneous Diseases,” is used to treat damp-heat jaundice with interior sthenia syndrome. In DXT, anthraquinones and alkaloids form insoluble complexes, reducing its effectiveness. A revised herbal extract, DXT-M, was developed, and its hepatoprotective properties were demonstrated in animal models using pharmacodynamic, metabolomic, network pharmacological, and toxicological approaches. **Methods**: The α-naphthalene isothiocyanate was utilised to establish the acute liver injury rat model. The assays of glutamate pyruvate transaminase, glutamic oxalacetic transaminase, alkaline phosphatase, bilirubin, total bile acid, complement 3 (C3) and C4, interleukin-2 (IL-2) and IL-6, tumour necrosis factor α (TNF-α), and pathological morphology were used to evaluate the hepatoprotection of DXT in comparison to DXT-M. The ^1^H-NMR-based serum and urine metabolomics were performed to identify potential biomarkers and metabolic pathways of DXT-M in treating hepatitis. The intrinsic regulatory mechanisms of DXT in liver protection, as well as the combination of network toxicology, were elucidated. Statistical analyses included RM two-way ANOVA with Geisser–Greenhouse correction and Dunnett’s post hoc test for longitudinal data, and one-way ANOVA with Dunnett’s post hoc test for group comparisons. Data were shown as mean ± SD. **Results**: Liver-injured animals exhibited weight loss, ruffled fur, and liver damage, accompanied by elevated liver function indicators. DXT-M effectively improved these symptoms, repaired liver damage, restored liver function, and regulated immune status by modulating complement 3. Metabonomics and other analyses indicated the CYP/GST-ROS axis is key to its hepatoprotective effects. DXT-M outperformed DXT in efficacy. **Conclusions**: DXT-M demonstrated significant effectiveness in restoring liver pathological damage, correcting abnormal biochemical indicators of liver function, and regulating complement factors. The pathway of CYP/GST-ROS served as the shared regulatory axis and transformation site for DXT-M’s liver protective effects. These findings suggest that DXT-M has potential as a treatment for acute liver injury, highlighting the need for further research into its underlying molecular mechanisms as well as its complete material basis. This study’s main limitation is its focus on acute models; future research should include other liver diseases and clinical observation to evaluate its full potential.

## 1. Introduction

As a vital organ, the liver performs essential functions including metabolism, detoxification, and immune regulation. Liver diseases—from acute injury to chronic conditions such as hepatitis, fibrosis, and cancer—pose significant global health burdens, causing millions of deaths annually and representing a leading cause of disability worldwide [1,2]. Acute Liver Injury (ALI) is a severe clinical syndrome marked by rapid hepatocyte necrosis, inflammatory pathway activation, and metabolic dysfunction, which can progress to fatal liver failure [3,4]. Elevated alanine aminotransferase (ALT), often triggered by hepatotoxic agents, viral infections, or autoimmune reactions, is a standard indicator of ALI [5,6]. The underlying mechanisms involve direct cytotoxicity leading to necrosis or apoptosis of hepatic and biliary epithelial cells, oxidative stress with glutathione depletion, immune activation and pro-inflammatory cytokine release, and dysregulated metabolic pathways—especially those related to energy metabolism and the tricarboxylic acid cycle [7,8]. Although advances have been made in understanding ALI pathogenesis, treatment remains limited, relying mainly on supportive measures and removal of causative factors [6,9]. Thus, there is a pressing need for effective multitarget pharmacological interventions. In this context, Traditional Chinese Medicine (TCM), characterised by multi-component formulations, presents a promising therapeutic paradigm [10,11]. By harmonising the “Yin–Yang” equilibrium within the body and enhancing the immune system, TCM exhibits preventive and therapeutic capabilities in disease management [12,13]. However, the accurate elucidation of the pharmacological basis of TCM is challenging due to variations in the types and quantities of chemical components, which mutually influence one another, thereby presenting difficulties in evaluating its efficacy and researching its mechanism of action [14,15]. The inconsistent quality of TCM raw materials, stemming from their diverse variety, origin, and variations in planting, collecting, and processing, poses a challenge in ensuring the stability and controllability of the herbal extracts, which introduce numerous uncertainties in clinical efficacy [16,17,18]. By employing extraction and refining preparative methods in TCM chemistry, the identification of active chemical constituents in refined TCM components has become relatively transparent, ensuring the stability, controllability, and clinical effectiveness of TCM components [19].

In TCM mixtures with hepatoprotective activity, many herbs contain carboxyl acids and phenolic compounds, exhibiting varying acidity levels. These herbs, which include specific constituents, are commonly referred to as acidic herbs, such as Rhei Radix Et Rhizoma (RRR) and Glycyrrhizae Radix Et Rhizoma [20,21,22]. Similarly, there is also a substantial presence of herbs containing alkaloids. These herbs, which possess varying degrees of alkalinity, are also referred to as alkaline herbs, such as Phellodendri Chinensis Cortex (PCC), Coptidis Rhizoma, and Aconiti Lateralis Radix Praeparata [23,24,25]. The paired herb of acidic and alkaline herbs in the same formula is known as acid–alkaline paired herbal medicine [26,27]. During the decocting process of TCM mixtures containing paired acid–alkaline herbs, such as those with anthraquinone and acid saponin, the acid components can combine with alkaloids to form insoluble complexes [28]. This phenomenon leads to the loss of active ingredients, ultimately affecting clinical effectiveness. The herbal prescription known as Dahuang Xiaoshi Tang (DXT), described in “Treatise on Febrile and Miscellaneous Diseases” (<<Shanghan Zabing Lun>> in China at 220 AD), is composed of Rhei Radix et Rhizoma (RRR), Phellodendri Chinensis Cortex (PCC), Gardeniae Fructus (GF), and Natrii Sulfas (NS). It is commonly used to treat damp-heat jaundice accompanied by interior sthenia syndrome, and different types of hepatitis caused by dampness and heat [29,30]. Its therapeutic potential against damp-heat jaundice is attributed to the integrated actions of key bioactive constituents, including anthraquinones from RRR [31,32,33,34], alkaloids such as berberine from PCC [35,36], and iridoid glycosides like geniposide from GF [37,38]. Interestingly, DXT is a classic example of a herbal formula featuring paired acid–alkaline herbs, specifically RRR-PCC [39]. During the decoction process of DXT, the anthraquinones in RRR readily bind with the alkaloids in PCC, forming an insoluble macromolecular complex that precipitates [40], resulting in the depletion of its active constituents and a decrease in the therapeutic effectiveness of DXT.

**Figure 1:**
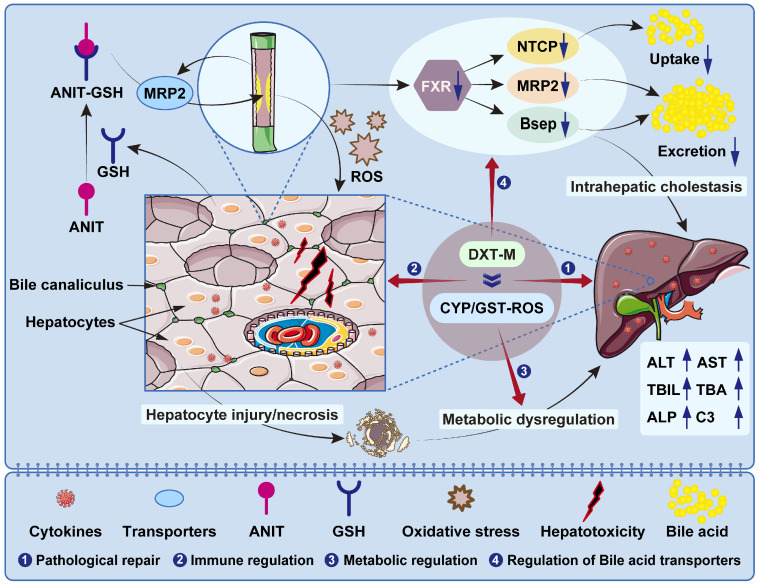
The proposed mechanisms of DXT-M in acute liver injury [41,43,44]. The blue arrows: upward indicated an increase or rise, while downward signified a decrease or reduction.

To address the reduction of active ingredients caused by the co-decoction of acidic and alkaline ingredients in DXT, a re-formulation approach was adopted to study the hepatoprotective efficacy and related mechanisms of DXT. Here, extracts from individual herbs, i.e., RRR, PCC, and GF, of DXT were prepared and combined according to an optimised proportion [41]. This revised preparation of DXT was named DXT-M. Based on the critical role of the liver in metabolic regulation, we speculate that DXT-M can improve acute liver injury by repairing endogenous metabolic disorders, immune imbalance and pathological damage. The proposed mechanisms of DXT-M in countering the pathogenesis of acute liver injury were summarised in Figure 1. In this study, we systematically evaluated the hepatoprotective effects of DXT-M in an ANIT-induced rat model of ALI through integrated pharmacological, metabolomic, network pharmacological, and toxicological analyses [42]. This study systematically assesses the hepatoprotective effects of the reformed DXT-M preparation. The primary objectives were to confirm DXT-M’s superior efficacy over traditional DXT, identify metabolic biomarkers and pathways affected by DXT-M using ^1^H-NMR-based metabolomics, and explore mechanisms and potential hepatotoxic risks through a “compound–target–pathway” network. This multi-omics approach seeks to provide a scientific basis for developing DXT-M as a treatment for acute liver injury.

## 2. Results

### 2.1. Chemical Analysis of DXT Herbal Extract

As illustrated in Supplementary Figure S1A, the HPLC chromatographic peaks of refined extracts deriving from RRR, PCC, and GF exhibited greater peak clarity than the crude herbal extract, while retaining the principal components. In alignment with the previous reports [22,45,46,47], representative anthraquinone substances were detected in crude and refined RRR extracts [31,32,33,34]. Similarly, berberine, a predominant alkaloid, was identified in both crude and refined extracts of PCC [35,36], and geniposide, a prevalent iridoid glycoside, was found in both crude and refined extracts of GF [37,38]. The structures of representative phytochemicals in the constituent herbs of DXT were presented in Supplementary Figure S1B. These compounds are representative of the major bioactive classes (anthraquinones, alkaloids, and iridoid glycosides) that underpin the pharmacological profile of DXT (Supplementary Table S1). Consequently, the material basis of the refined extract of each herb in DXT-M, achieved by eliminating impurities and retaining essential constituents, was more clearly defined than that of the crude extracts. This refinement enhances quality, stability and controllability, providing a foundation for subsequent efficacy determination.

### 2.2. Effect of DXT and DXT-M on Animals with Liver Damage

**Figure 2:**
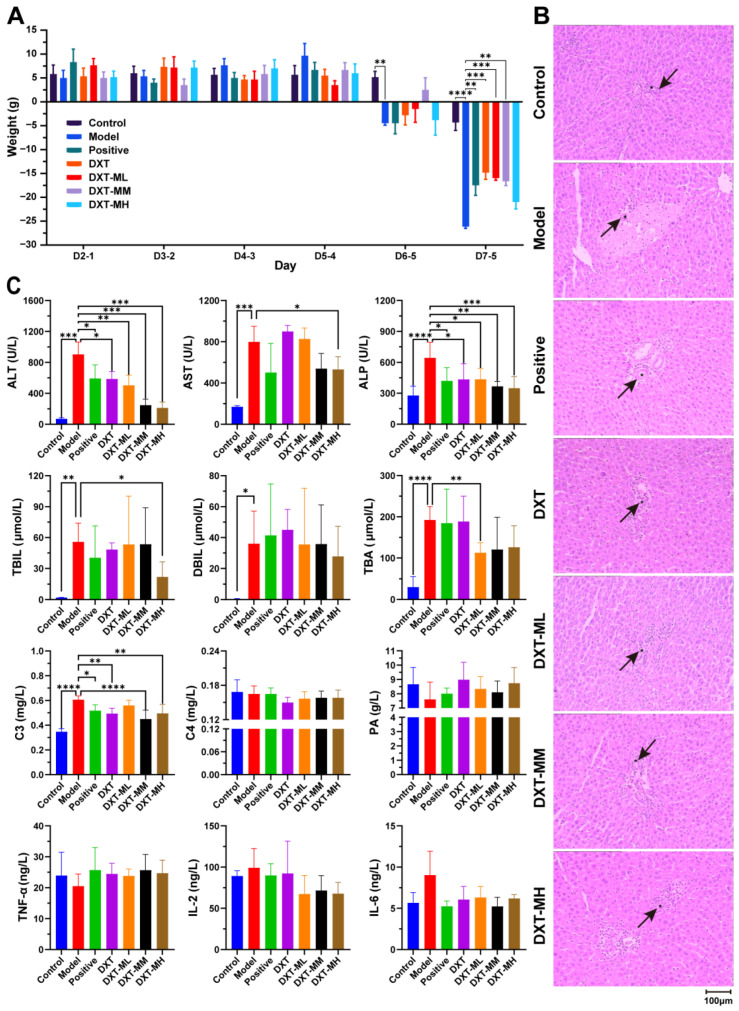
Effects of DXT and DXT-M on animals with liver damage. (**A**) Therapeutic effects of DXT and DXT-M on sequential body weight changes in ANIT-induced liver injury rats. X-axis: consecutive day differences (ΔDn = Dn − Dn-1, where Dn is body weight on day n); Y-axis: data are expressed as mean ± SEM. Statistical analysis was performed using Geisser–Greenhouse corrected RM two-way ANOVA followed by Dunnett’s post hoc test (** *p* < 0.01, *** *p* < 0.001, and **** *p* < 0.0001 vs. ANIT model group). (**B**) Representative *H&E*-stained liver sections from experimental groups (100× magnification). The black arrows symbolise the biliary duct. Scale bar: 100 μm. (**C**) Effects of DXT and DXT-M on serum levels of liver function markers (ALT, AST, ALP), bilirubin (TBIL), total bile acids (TBA), complement components (C3, C4), and inflammatory cytokines (TNF-α, IL-2, IL-6) in ANIT-induced liver injury rats. Data are expressed as mean ± SD (*n* = 6 per group). Statistical significance determined by one-way ANOVA with Dunnett’s post hoc test (* *p* < 0.05, ** *p* < 0.01, *** *p* < 0.001, and **** *p* < 0.0001 vs. ANIT model group).

Qualitative observations (data not presented) revealed that, compared to the control group, animals with a-naphthylisothiocyanate (ANIT)-induced liver injury showed reduced activity, feeding, and body weight, and exhibited recumbency, yellow urine, and perianal soiling. After receiving therapeutic agents, these symptoms improved to different extents. The DXT and low-dose DXT-M groups showed increased vigour, while the medium and high-dose DXT-M groups significantly improved recumbency, perianal soiling, and yellow urine, as well as vigour. As depicted in Figure 2A, all groups gained weight consistently before liver injury. However, on the sixth day, the animals with liver injury experienced significant weight loss compared to the controls. DXT-treated groups showed less weight reduction, with the medium-dose DXT-M group showing recovery. By the seventh day, all groups experienced weight loss. The model group showed a marked reduction compared to the control group, and, except for the high-dose DXT-M group, the body weight decline rate in the other treatment groups was significantly mitigated relative to the model group.

The histopathological analysis in Figure 2B showed that normal animals had well-structured liver lobules, intact hepatocytes, clear bile ducts, and no vein dilation or edema. In contrast, liver-injured animals displayed disrupted lobules, hepatocyte necrosis, portal inflammation, narrowed bile ducts, and dilated veins with stasis and edema. Treatment with DXT and various doses of DXT-M improved these tissue injuries to varying extents. In the decoction groups, hepatocyte necrosis decreased, and the bile duct structure improved, although the lumens were incomplete. In groups receiving different DXT-M doses, hepatocytes showed minimal necrosis, normal portal structure, reduced inflammation, clear bile ducts with intact epithelium, and normal veins. Figure 2C demonstrated that liver injury significantly raised liver function indices (ALT, AST, ALP, TBIL, TBA) compared to controls. Treatments varied in effectiveness, with ursodeoxycholic acid and DXT notably reducing ALT and ALP. Furthermore, DXT-M exhibited a favourable dose-dependent modulation of ALT and ALP, surpassing the efficacy of DXT. High-dose DXT-M significantly decreased AST and TBIL, while low-dose DXT-M lowered TBA. Liver-injured animals had higher complement C3 levels, which ursodeoxycholic acid, DXT, and medium/high doses of DXT-M normalised.

### 2.3. Regulation of DXT-M on Endogenous Metabolites of Animals with Liver Damage

#### 2.3.1. Metabolites Identification of Serum and Urine in 1H-NMR Spectrum

Here, the chemical shifts of peaks and coupled split peaks in the ^1^H-NMR spectra of serum (δ0.6–4.2) and urine (δ0.52–4.56 and δ6.0–9.4) from animals in each group were analysed. From the serum ^1^H-NMR spectra, eight differential metabolites were identified, including amino acids such as threonine, glutamic acid, taurine, glutamine, and their derivatives, as well as lipid metabolites and carbohydrate substances like glucose and myo-inositol. In the urine ^1^H-NMR spectra, eleven differential metabolites were identified, encompassing amino acids such as phenylalanine, glycine, homoserine, lysine, and alanine; organic acids such as oxoglutaric acid, citric acid, and aminoadipic acid; gut-host co-metabolites such as phenylacetylglycine and hippuric acid; and nitrogen-containing substances such as methylamine. The typical ^1^H-NMR spectra for serum and urine from each group of animals were depicted in Supplementary Figure S2, with the identification results of metabolites for each group provided in Supplementary Table S2.

#### 2.3.2. Chemometric Analysis of Serum and Urine 1H-NMR Data

**Figure 3:**
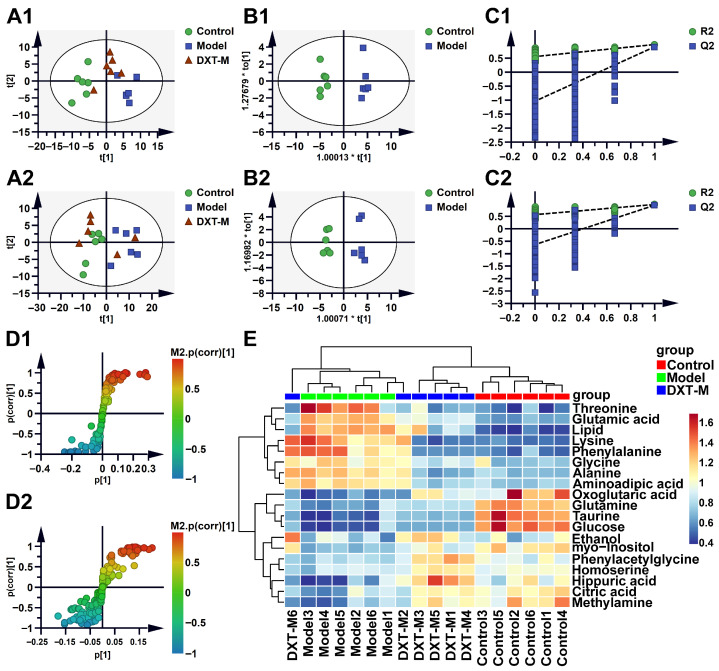
Multivariate analysis of serum and urine metabolomics. (**A1**–**D1**) Serum analysis: (**A1**) PCA score plots of Control, Model, and DXT-M treated groups; (**B1**) OPLS-DA model between Control vs. Model; (**C1**) Permutation test (200 iterations); (**D1**) s-Plot with *|p(corr)|* ≥ 0.5 highlighting key metabolites. (**A2**–**D2**) Corresponding urine analyses. *t*[1] and *t*[2] indicate the first and second principal components, respectively. The asterisk (*) at the axis labels (t[1], t[2]) denotes scaled scores in the OPLS-DA model. (**E**) Hierarchical clustering heatmap of significantly altered metabolites (red: upregulation; blue: downregulation; *p* < 0.05, *VIP* > 1.0). Heatmap columns: C1–C6 (Control), M1–M6 (Model), DXT-M1–6 (DXT-M).

PCA analysis of serum and urine metabolomic data in Figure 3(A1,A2) revealed distinct group separations: the control group on the left negative axis, the model group on the right positive axis, and the DXT-M group in between, suggesting its potential to balance metabolites in liver-injured animals. OPLS-DA in Figure 3(B1,B2) further classified these groups, showing significant differences with high R^2^Y and Q^2^ values for serum (0.985 and 0.903) and urine (0.976 and 0.945), indicating strong model interpretation and predictivity. Figure 3(C1,C2) showed that permutation tests (200 iterations) for serum and urine consistently resulted in lower left R^2^ and Q^2^ values compared to the original values on the right. Figure 3(D1,D2) S-plots identified key metabolites in a liver injury model, with variable importance indicated by *VIP* values. Hierarchical clustering in Figure 3E separated control and model groups. Following the intervention with DXT-M, the clustering pattern of the model group showed a tendency to revert to that of the control group. In comparison to the control group, the model group exhibited significantly elevated concentrations of metabolites, including oxoglutaric acid, glutamine, taurine, glucose, ethanol, myo-inositol, phenylacetylglycine, homoserine, hippuric acid, citric acid, and methylamine. In the DXT-M-treated group, the levels of these metabolites showed a trend similar to that observed in the control group. Similarly, the model group displayed significantly reduced levels of threonine, glutamic acid, lipid, lysine, phenylalanine, glycine, alanine, and aminoadipic acid compared to the control group. The levels of these metabolites in the DXT-M-treated group also exhibited a trend toward normalisation.

#### 2.3.3. Endogenous Screening and Correlation Analysis with Biochemical Indicators

**Figure 4:**
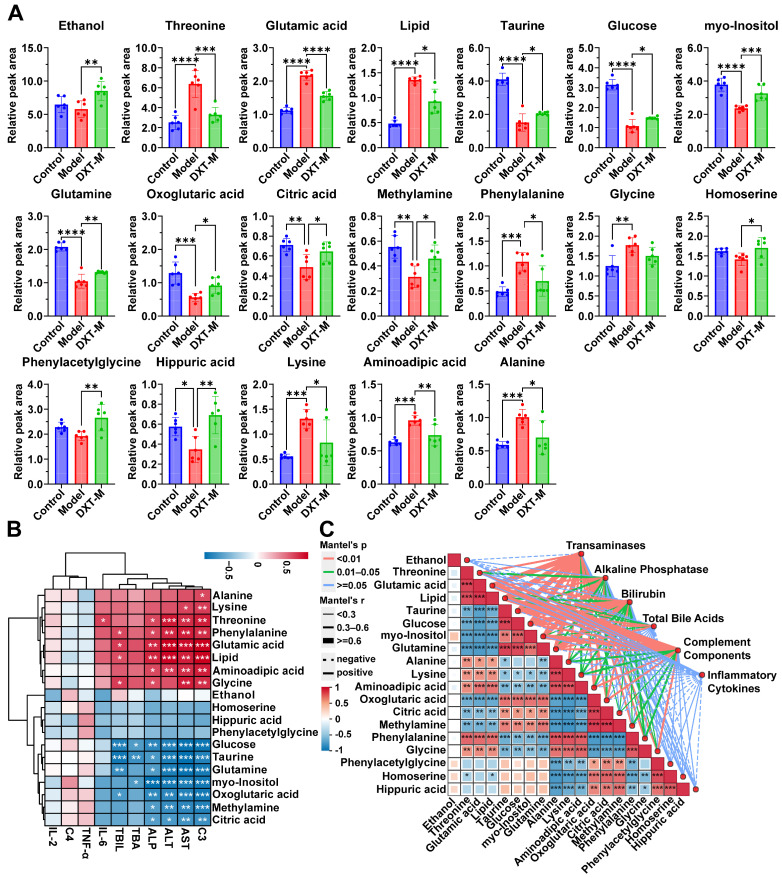
Metabolomic alterations and their pathophysiological correlations. (**A**) Relative peak areas of differential metabolites in serum and urine metabolomics across experimental groups. Data are presented as mean ± SD (*n* = *6* per group). Statistical significance was determined by one-way ANOVA followed by Dunnett’s post hoc test (* *p* < 0.05, ** *p* < 0.01, *** *p* < 0.001, and **** *p* < 0.0001 vs. ANIT model group). (**B**) Hierarchical cluster heatmap (complete linkage, Euclidean distance) of Spearman’s rank correlations between differential metabolites and clinical indices. |*r*| ≥ 0.6, and Benjamini–Hochberg FDR-adjusted *p* < 0.05. Analysed parameters include liver enzymes (ALT, AST, ALP), bilirubin (TBIL), total bile acids (TBA), complement components (C3, C4), and inflammatory cytokines (TNF-α, IL-2, IL-6). The color gradient indicates the strength of correlation (red: positive correlation, *r* > 0.6; blue: negative correlation, *r* < −0.6), with the intensity proportional to the magnitude of the coefficient. (**C**) Mantel test correlation matrix between clinical indices (transaminases, alkaline phosphatase, bilirubin, total bile acids, complement components, and inflammatory cytokines) and differential metabolites. Edge thickness reflects Mantel’s *r* statistic with 999 permutations (*p* < 0.05 by Benjamini–Hochberg FDR correction).

A univariate *t*-test was used to assess the significance of metabolites across the control, model, and DXT-M groups, identifying key metabolites via S-plot, *VIP* values (*VIP* > 1), and *t*-test (*p* ≤ 0.05). As shown in Figure 4A and Supplementary Table S2, 8 metabolites were identified as potential biomarkers in the serum. Compared to controls, taurine, glucose, myo-inositol, and glutamine levels decreased, except for ethanol, but were adjusted after DXT-M treatment. Threonine, glutamic acid, and lipid levels increased but normalised post-treatment. Similarly, 11 significant urinary biomarkers were identified. In liver-injured animals, urine levels of alanine, lysine, aminoadipic acid, phenylalanine, and glycine rose significantly compared to the control group. Treatment with DXT-M reduced these metabolites, with all except glycine showing significant changes. Conversely, oxoglutaric acid, citric acid, methylamine, phenylacetylglycine, homoserine, and hippuric acid levels decreased, with substantial reductions in all but phenylacetylglycine and homoserine. DXT-M treatment significantly increased the levels of these 11 metabolites.

The association between biochemical indicators and potential metabolites was analysed using Spearman correlation and the Mantel test, with the results shown in Figure 4B,C. Figure 4B revealed that alanine, lysine, threonine, phenylalanine, glutamic acid, lipid, aminoadipic acid, and glycine positively correlate with C3, AST, ALT, ALP, TBIL, TBA, and IL-6, while glucose, taurine, glutamine, myo-inositol, oxoglutaric acid, methylamine, and citric acid showed negative correlations. C3, AST, ALT, ALP, TBIL, TBA, and IL-6 clustered together, separated from TNF-α, C4, and IL-2. The heatmap in Figure 4C highlighted significant correlations: transaminases with taurine, glucose, glutamine, α-ketoglutaric acid, and amino adipic acid; alkaline phosphatase with inositol and glutamic acid; and bilirubin with taurine, glucose, and glutamine. Total bile acids were only correlated with taurine and glucose. Complement components negatively correlated with lipids, glutamic acid, inositol, and threonine. These results, consistent with Figure 4B, suggested that biochemical changes were associated with variations in metabolite levels. The Mantel test showed significant spatial correlations among metabolites. Threonine, glutamic acid, and lipids positively correlated with alanine, lysine, and aminoadipic acid, but negatively with taurine, glucose, myo-inositol, and glutamine. TCA cycle metabolites, oxoglutaric and citric acid, were synergistic with methylamine, positively correlating with homoserine and hippuric acid, and negatively with phenylalanine. Phenylacetylglycine and homoserine also showed synergy, positively correlating with hippuric acid and negatively with alanine.

#### 2.3.4. Analysis of the Metabolic Pathway

**Figure 5:**
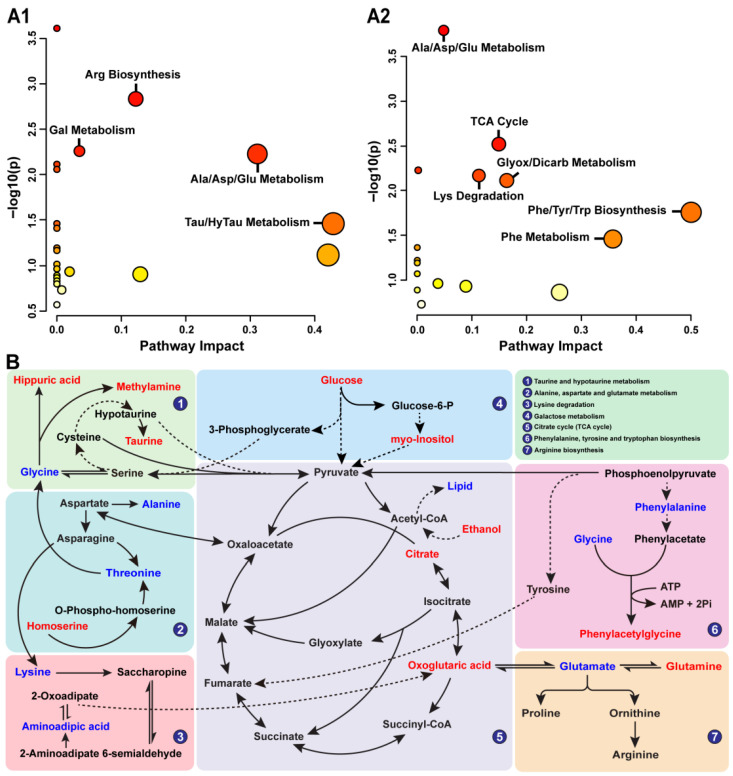
Metabolic pathway enrichment and interaction network of differential metabolites. (**A1**) Serum and (**A2**) urine metabolomics pathway enrichment analysis. Bubble colour intensity represents −log10(p) values (red: higher significance); bubble size indicates pathway impact value (larger size = greater regulatory effect). (**B**) Interaction network between annotated differential metabolites and metabolic pathways. Metabolites in red were significantly upregulated by DXT-M treatment, while those in blue were downregulated. Solid lines: direct interactions; dashed lines: indirect interactions. Key enriched pathways: ① Taurine and hypotaurine metabolism, ② Alanine, aspartate and glutamate metabolism, ③ Lysine degradation, ④ Galactose metabolism, ⑤ Citrate cycle (tricarboxylic acid cycle, TCA cycle), ⑥ Phenylalanine, tyrosine and tryptophan biosynthesis, ⑦ Arginine biosynthesis.

Key metabolites in serum and urine regulated by DXT-M were analysed using MetaboAnalyst 5.0 to investigate their corresponding metabolic pathways. Pathways with an impact value ≥ 0.01 and *p*-value ≥ 1 were considered relevant to DXT-M’s liver protection. Serum metabolites enriched pathways like arginine biosynthesis, galactose metabolism, alanine, aspartate and glutamate metabolism, taurine and hypotaurine metabolism (Figure 5(A1)), while urine metabolites enriched pathways such as the alanine, aspartate and glutamate metabolism, phennylalanine metabolism, TCA cycle, glyoxylate and dicarboxylate metabolism, lysine degradation, phenylalanine metabolism, phenylalanine, tyrosine and tryptophan biosynthesis (Figure 5(A2)). In addition, it is worth noting that alanine, aspartate and glutamate metabolism were the common metabolic pathways enriched in both serum and urine, indicating their critical role in managing liver dysfunction by DXT-M. In addition, a KEGG-based metabolic network diagram depicted in Figure 5B highlighted the involvement of metabolites like hippuric acid, methylamine, taurine, and others in pathways related to taurine and hypotaurine metabolism, alanine, aspartate and glutamate metabolism, lysine degradation, galactose metabolism, the tricarboxylic acid (TCA) cycle, and biosynthesis of phenylalanine, tyrosine, tryptophan, and arginine.

### 2.4. Integrated Analysis of Hepatoprotective Mechanisms

#### 2.4.1. Screening Results of Potential Targets

**Figure 6:**
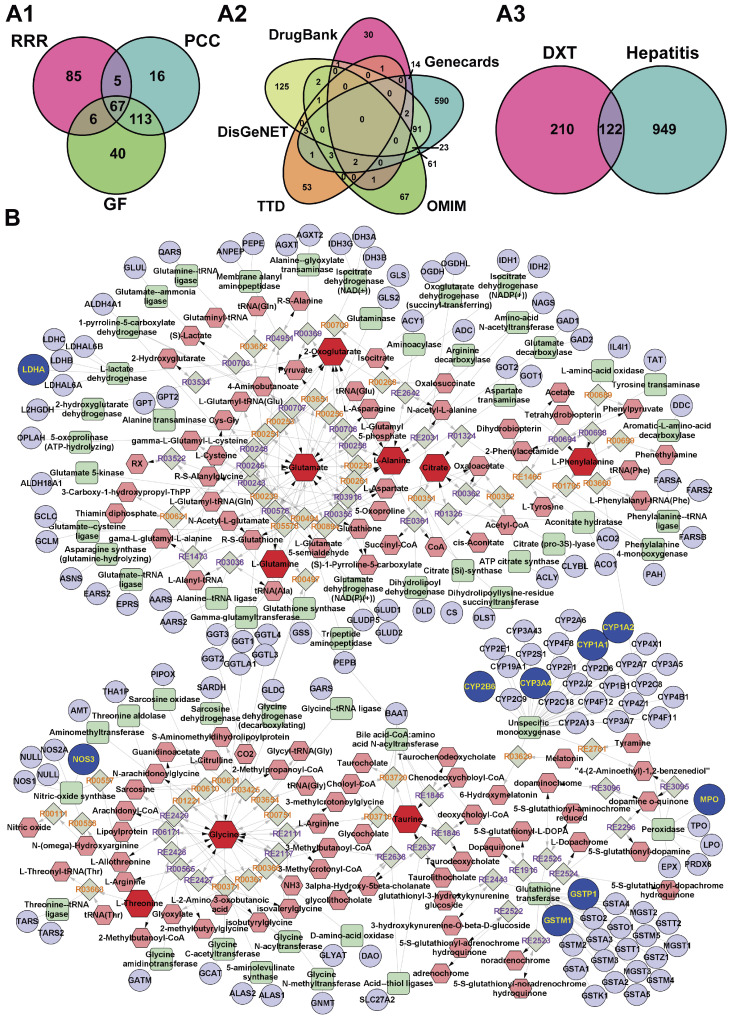
Venn analysis and compound–reaction–enzyme–gene (CREG) network of DXT-M in hepatitis treatment. (**A1**) Venn diagram of potential targets for DXT-M from TCMSP database. (**A2**) Hepatitis-related targets collected from DrugBank, DisGeNET, GeneCards, TTD, and OMIM databases. (**A3**) Intersection targets between DXT-M and hepatitis. (**B**) CREG network integrating DXT-M and hepatitis shared targets with differential metabolites from serum/urine metabolomics. Node types: red hexagons (compounds), gray diamonds (reactions), green squares (enzymes), blue circles (genes). Darker colors highlight key nodes: deep red (significantly altered metabolites), deep blue (DXT-M and hepatitis intersection targets).

Using the TCMSP database, the chemical constituents of DXT-M were analysed, identifying 322 unique targets after merging duplicates (Figure 6(A1)). An OMIM search for “hepatitis” found 171 targets, while GeneCards provided 790 targets with high relevance scores. Additional targets were sourced from TTD (83), DisGeNET (248), and DRUGBANK (79), resulting in a comprehensive database of 1017 disease targets (Figure 6(A2)). The Venny platform identified 122 overlapping targets between DXT-M and hepatitis (Figure 6(A3)). These were further analysed using the Metscape plug-in v3.1.3 in Cytoscape v3.8.2, revealing 37 targets in the metabolite–reaction–enzyme–gene network.

#### 2.4.2. Compound-Reaction-Enzyme-Gene Network

As illustrated in Figure 6B, the advanced integrated analysis conducted by Metscape has successfully generated networks comprising compounds, reactions, enzymes, and genes across multiple metabolic pathways, including bile acid biosynthesis, various amino acid metabolisms, glycolysis, gluconeogenesis, and the TCA cycle, among others. The target enzyme LDHA is involved in glycolysis and gluconeogenesis by interacting with endogenous metabolites such as ethanol and glucose. Targets such as MPO and GSTM1 are implicated in the regulation of tyrosine metabolism through phenylalanine. GSTM1, GSTP1, NOS3, and other enzymes modulated the urea cycle and associated amino acid metabolisms through endogenous metabolites. Enzymes such as GPT and ODC1 also influence these metabolic processes via distinct endogenous metabolites. Furthermore, GSTM1, CYP1A1, and other enzymes regulate tryptophan metabolism through enzyme-mediated regulation of metabolites. Glycine and taurine are potentially involved in bile acid biosynthesis through indirect target regulation, while glycine, alanine, and threonine participate in their respective amino acid metabolisms. Citrate and 2-oxoglutarate are likely involved in the TCA cycle. These metabolites and targets are interconnected through shared or key regulatory nodes, forming an integrated molecular network characterised by hierarchical synergy. This network shows coordination between phenotypic effects and intrinsic mechanisms.

#### 2.4.3. Target Interaction Analysis

**Figure 7:**
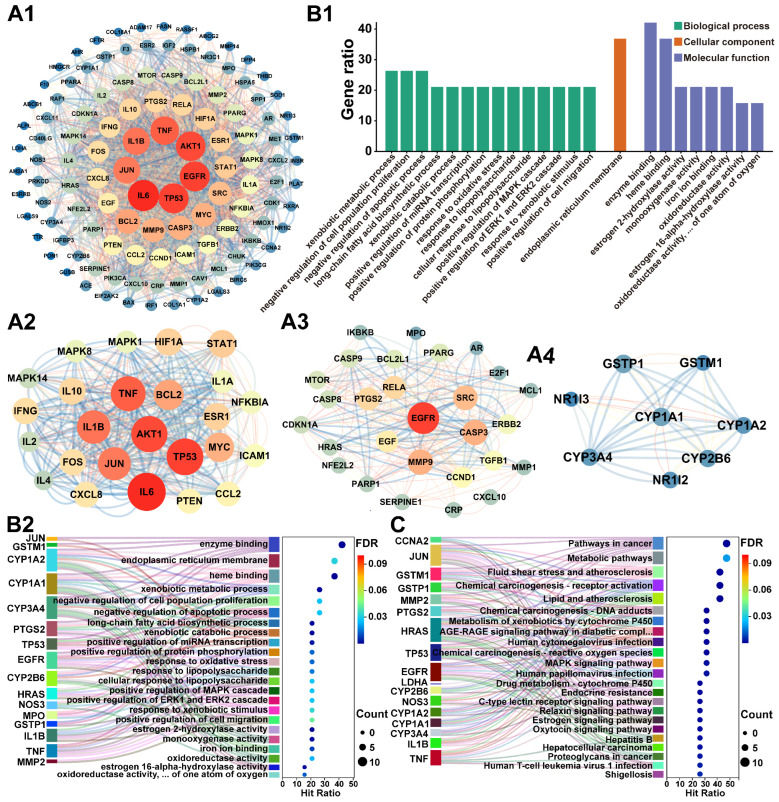
Network pharmacological analysis of DXT-M in hepatitis intervention. (**A1**–**A4**) Protein–protein interaction (PPI) network analysis showing: (**A1**) Global PPI network of 122 intersection targets. (**A2**–**A4**) Topologically significant subnetworks identified via MCODE plug-in in Cytoscape (v3.8.2); Cluster 1 (Score: 18.083, 25 nodes), Cluster 2 (Score: 9.481, 28 nodes), Cluster 3 (Score: 6.571, 8 nodes), with node colour/size indicating degree centrality and edge thickness reflecting STRING combined scores. (**B1**,**B2**) Functional enrichment analysis including: (**B1**) Significantly enriched GO terms (FDR-adjusted *p* < 0.05) displayed as a clustered bar plot; (**B2**) Sankey-bubble plot displaying left DXT-M targets, middle GO terms, and right enrichment bubbles. (**C**) KEGG pathway enrichment Sankey-bubble plot. The layout follows the same scheme as (**B2**), with the right column showing enriched KEGG pathways. The GO term “oxidoreductase activity, … of one atom of oxygen” refers to “oxidoreductase activity, acting on paired donors, with incorporation or reduction of molecular oxygen, reduced flavin or flavoprotein as one donor, and incorporation of one atom of oxygen”. The KEGG pathway “AGE-RAGE signalling pathway in diabetic compl” refers to the AGE-RAGE signalling pathway in diabetic complications.

The 122 potential targets of DXT-M for hepatitis treatment, as outlined in Section 2.4.1, were imported into the STRING database with the species parameter set to ‘human’ and the screening criterion established at a high-confidence threshold of 0.700 for PPI analysis. Subsequently, the data were imported into Cytoscape v3.8.2 software to construct a PPI network and perform topological analysis, as depicted in Figure 7(A1). Utilising this framework, the MCODE plug-in was employed to identify three core sub-networks within the PPI network, illustrated in Figure 7(A2–A4). Analysis of the core sub-networks in Figure 7(A2,A3) reveals that the key targets with higher degree values essentially correspond to the principal targets within the PPI network of the 122 potential DXT-M targets for hepatitis treatment. Furthermore, examination of the core sub-network in Figure 7(A4) indicates that nodes such as GSTP1, GSTM1, CYP1A1, CYP1A2, CYP2B6, and CYP3A4 exhibit a significant degree of overlap with the integrated targets identified in Section 2.4.2. Consequently, based on the degree values of each target in Figure 7(A1), and in conjunction with the representative component targets of DXT-M and the integrated targets from Section 2.4.2, a total of 19 targets were selected (see Supplementary Table S3).

#### 2.4.4. Hepatoprotective Targets Enrichment Analysis

Nineteen potential targets were analysed with the DAVID database for GO and KEGG enrichment, focusing on FDR-adjusted *p*-values (*p* < 0.05) and Count values. This identified 14 BP terms (Count ≥ 4), 1 CC term, and 8 MF terms (Count ≥ 3), as shown in Figure 7(B1). BP terms included xenobiotic metabolism, cell proliferation, and apoptosis regulation, among others. The CC term was about the endoplasmic reticulum membrane, and MF terms covered enzyme, heme binding, etc. Correlation analysis (Figure 7(B2)) revealed a regulatory network centred on the CYP450 enzyme family (CYP1A2, CYP1A1, CYP3A4, CYP2B6), which is involved in xenobiotic metabolism and other activities. CYP1A2, CYP1A1, and CYP3A4 also participate in long-chain fatty acid biosynthesis. PTGS2, MPO, etc., are associated with oxidative stress responses and affect apoptosis and cell proliferation, contributing to the MAPK/ERK cascade. Protein interaction analysis revealed that CYP enzymes interacted with JUN, among others, and some responded to lipopolysaccharide. KEGG pathway analysis (Figure 7C) revealed that CYP1A2, among others, was enriched in xenobiotic metabolism. CYP3A4 was involved in all metabolism pathways, and CYP2B6 was linked to lipid metabolism and atherosclerosis. HRAS, among others, was central in cancer pathways. NOS3, etc., controlled cardiovascular disease pathways, and HRAS, etc., played a key role in hormone signalling networks.

### 2.5. Network Toxicology Analysis

#### 2.5.1. Target Screening and Interaction Analysis

**Figure 8:**
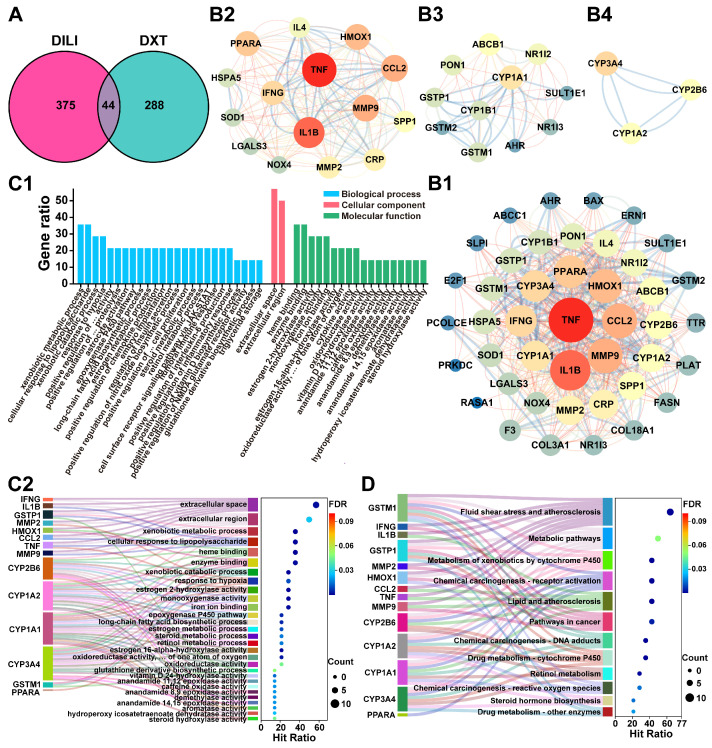
Network toxicological of DXT-M associated drug-induced liver injury (DILI) risk. (**A**) Venn diagram of overlapping targets between DXT-M and known DILI-related targets. (**B1**–**B4**) PPI network analysis: (**B1**) Global PPI network of 44 intersection targets; (**B2**–**B4**) Topologically significant modules identified via MCODE in Cytoscape (v3.8.2)-Cluster 1 (Score: 12.714, 15 nodes), Cluster 2 (Score: 6.6, 11 nodes), Cluster 3 (Score:3.0, 3 nodes), with node properties scaled by degree centrality and edge weights reflecting STRING confidence scores. (**C1**,**C2**) Functional annotation: (**C1**) Significantly enriched GO terms (FDR-adjusted *p* < 0.05) categorised by biological processes, cellular components and molecular functions; The GO term “positive regulation of … proteolysis” refers to the complete term: “positive regulation of membrane protein ectodomain proteolysis”. (**C2**) Sankey-bubble plot visualising target–GO term relationships with bubble size representing target count and colour indicating FDR *p*-value. (**D**) KEGG pathway enrichment Sankey-bubble plot. The GO term “positive regulation of … activity” refers to “positive regulation of calcidiol 1-monooxygenase activity”; The GO term “positive regulation of … proteolysis” refers to “positive regulation of membrane protein ectodomain proteolysis”; The GO term “positive regulation of … cell proliferation” refers to “positive regulation of vascular associated smooth muscle cell proliferation”; The GO term “oxidoreductase activity, … of one atom of oxygen” refers to “oxidoreductase activity, acting on paired donors, with incorporation or reduction of molecular oxygen, reduced flavin or flavoprotein as one donor, and incorporation of one atom of oxygen”.

The Comparative Toxicogenomics Database identified 419 targets linked to drug-induced liver injury. After combining with 322 DXT-M targets, a Venn diagram showed 44 overlapping targets (Figure 8A). These were analysed using the STRING database and Cytoscape v3.8.2 to create a PPI network, as shown in Figure 8(B1). The MCODE plug-in identified core subnetworks of DXT-M liver injury targets, depicted in Figure 8(B2–B4). Key targets, such as CYP3A4, CYP1A2, CYP2B6, CYP1A1, GSTP1, and GSTM1, appear in both the PPI network and core subnetworks, and are crucial for DXT-M’s role in modulating metabolism, as detailed in Section 2.4.2. Concurrently, TNF, IL1B, CCL2, MMP9, HMOX1, PPARA, and IFNG were key nodes in the PPI network and core subnetwork. Consequently, 14 core liver toxicity targets of DXT-M were identified for further analysis (Supplementary Table S4).

#### 2.5.2. Toxicity-RelatedTargets Enrichment Analysis

The 14 toxic targets of DXT-M from Section 2.5.1 were analysed using the DAVID database for GO and KEGG pathway enrichment analysis. With FDR-adjusted *p*-value (*p* < 0.05), 14 BP, 2 CC, and 19 MF terms were identified (Figure 8(C1)). Key BP terms included xenobiotic, lipid, and metabolism, as well as cellular stress and immune responses. Other BP terms involved signalling and enzymatic activities, highlighting vascular and inflammatory regulation. The CC terms, extracellular space and region, linked targets to the extracellular compartment. Enriched MF terms mainly involved enzymatic catalytic functions, especially oxidoreductase activities, and binding functions. Similar to DXT-M pharmacodynamic targets, CYP is central to DXT-M toxicity targets and GO entries. Figure 8(C2) showed that toxicity targets were enriched in xenobiotic metabolism (CYP1A2, CYP1A1, CYP3A4, CYP2B6, GSTM1, GSTP1), extracellular localisation (IFNG, IL1B, TNF, MMP9), and heme/iron-dependent catalysis, with key activities contributing to stress responses and lipid/steroid metabolism. Figure 8D indicated that KEGG pathway enrichment analysis identified key drug toxicity targets, including CYP1A2, CYP3A4, GSTM1, and GSTP1, mainly in drug metabolism pathways. HMOX1 was linked to fluid shear and oxidative stress pathways. IL1B, TNF, etc., were associated with inflammation and cancer-related pathways. PPARA was involved in the regulation of chemical carcinogenesis and inflammatory response pathways.

### 2.6. Target–Component Fitting Validation

**Figure 9:**
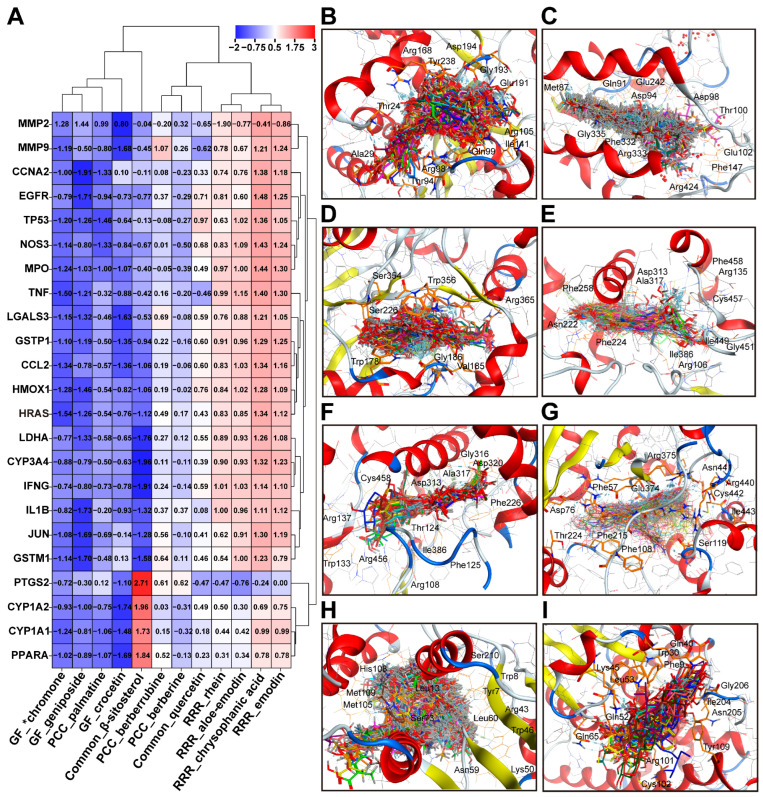
Fitting validation of DXT-M representative compounds with core targets. (**A**) Hierarchically clustered heatmap (complete linkage, Euclidean distance) of docking scores between 12 representative DXT-M compounds and 23 integrated targets (network pharmacology–toxicology screening). Data were column-wise Z-score normalised (blue: strong binding affinity; red: weak affinity). (**B**–**I**) Ligand–receptor binding conformations: Ligands shown as stick models (colored by atom type), proteins as molecular surfaces (electrostatic potential). PDB IDs: LDHA (5W8J), MPO (4C1M), NOS3 (7TSG), CYP1A1 (4I8V), CYP1A2 (2HI4), CYP3A4 (6MA8), GSTM1 (8VOU), and GSTP1 (AF-P09211-F1).

Receptor proteins like EGFR, TP53, and TNF were sourced from the PDB, while GSTP1, CCL2, and IFNG were obtained from UniProt. DXT-M drugs contain anthraquinones, iridoid glycosides, and alkaloids, forming a chemical library of bioactive constituents. Active sites of targets, including EGFR and TP53, were identified using co-crystallised ligands, while proteins without such ligands were used MOE’s Site Finder for pocket identification. HRAS interactions were examined through full-protein docking. Binding Z-scores are in Supplementary Table S5 and Figure 9A, with interaction diagrams in Figure 9B–I and PLIF analysis in Supplementary Figure S3. In the ligand–target fitting score clustering diagram (Figure 9A), DXT-M active ingredients were grouped into anthraquinones from RRR, iridoid glycosides from GF, and alkaloids from PCC. One target cluster included PTGS2, CYP1A2, CYP1A1, and PPARA, while others formed a separate cluster. Anthraquinones in RRR showed high affinity for the first cluster but lower scores. Geniposide, crocin from GF, and palmatine in PCC had high affinity with 23 targets, whereas berberine and berberrubine in PCC had weaker affinity. Anthraquinones in RRR mainly targeted PTGS2, CYP1A2, CYP1A1, and PPARA. β-sitosterol from RRR, PCC, and GF bound well to most targets, except for PTGS2, CYP1A2, CYP1A1, and PPARA. Quercetin showed weak affinity for all targets. The 3D interaction analysis revealed key binding residues: LDHA (Arg168, Glu191, His192, Asp194), MPO (Asp94, His95, Asp98, Thr100), NOS3 (Cys184, Trp156), CYP1A1 (Asn255, Phe258, Asp313, Ala317), CYP1A2 (Thr124, Phe260, Gly316, Ala317, Asp320), CYP3A4 (Arg105, Arg212, Arg372, Glu374, Arg375), GSTM1 (Tyr7, Trp8, Ser73), and GSTP1 (Arg14, Leu53, Tyr109).

### 2.7. The Component–Target–Pathway Association Analysis

**Figure 10:**
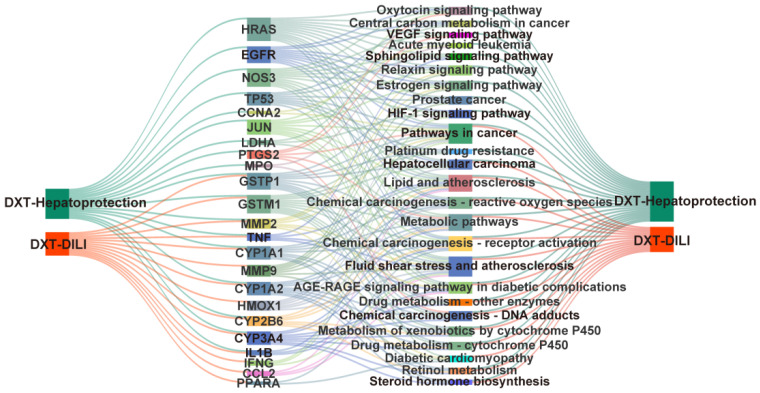
Systems pharmacology network of DXT-M dual effects on liver injury.

After integrating and deduplicating DXT-M’s targets, 24 unique targets were identified. Enrichment analysis via the DAVID Database revealed 25 significant pathways, excluding LDHA. Figure 10 illustrates the hepatoprotective and hepatotoxic relevance of DXT-M through shared targets, including GSTM1, IL1B, GSTP1, MMP2, TNF, CYP2B6, CYP1A1, CYP1A2, and CYP3A4. These targets may function through common pathways related to metabolism, cytochrome P450, xenobiotic metabolism, retinol metabolism, steroid hormone biosynthesis, vascular issues, atherosclerosis, and cancer, including hepatocellular carcinoma and chemical carcinogenesis. DXT-M’s liver-protective effects may involve targets such as JUN, NOS3, and TP53, which function through relaxin, estrogen, and HIF-1 signalling pathways, and may also influence cancer-related pathways, including prostate cancer and VEGF signalling. In contrast, its liver toxicity is linked to targets like IFNG and HMOX1, mainly through the diabetic cardiomyopathy pathway, potentially increasing risks due to overlapping pathways.

## 3. Discussion

### 3.1. Metabolic Reprogramming Underlies DXT-M’s Hepatoprotective Mechanisms

TCM’s holistic approach and syndrome differentiation are fundamental to its diagnostic and therapeutic efficacy, and the metabolomic profile identified in this study objectively reflected the disease phenotype, aligning with the holistic principles of TCM [48,49]. Prescriptions like DXT, formulated through component-based compatibility and refined constituent retention, demonstrate enhanced pharmacological precision. DXT-M significantly improved hepatoprotective indicators—notably body weight, serum enzymes (ALT, AST), bilirubin, bile acids, and complement C3—while repairing liver damage with a clear dose–response relationship, outperforming traditional decoction [43,44,50]. While precision medicine advances target identification, its single-target paradigm often fails to address the complex heterogeneity of diseases [51]. TCM syndrome, reflecting systemic regulatory imbalance, aligns with metabolomics profiling of endogenous biomarkers. This study identified 19 serum/urine biomarkers, with liver injury exhibiting dysregulation in metabolites, including glutamic acid, alanine, taurine, and hippuric acid—many of which are linked to TCA cycle-driven dysmetabolism of glucose, lipids, and amino acids [52,53]. The correlation network delineated in Figure 5B provides a mechanistic roadmap linking these metabolic alterations modulated by DXT-M to the observed phenotypic recovery. Several key correlations warrant elaboration. First, the strong negative correlations between taurine, glucose, glutamine, and critical liver injury indices (ALT, AST, TBIL, TBA) are highly informative. As a key hepatoprotective agent, taurine alleviates cholestasis by conjugating bile acids (reducing TBIL/TBA) [54,55] and mitigates hepatocyte damage via antioxidant activity (lowering ALT/AST) [56,57,58]. In addition, taurine supports fat and cholesterol metabolism [59,60]; its depletion during liver injury impairs these functions, a deficit that DXT-M treatment effectively counteracts.

The negative correlation of glucose and glutamine with these enzymes underscores the critical role of energy metabolism in liver repair. DXT-M’s restoration of glucose and glutamine levels likely replenishes the tricarboxylic acid (TCA) cycle, providing essential energy and biosynthetic precursors for hepatocyte regeneration, which in turn normalises transaminase levels. Furthermore, the positive correlations of amino acids such as alanine, lysine, and threonine with the same panel of injury markers underscore their potential as biomarkers of disrupted nitrogen metabolism. In a damaged liver, impaired transamination and ureagenesis lead to the accumulation of these amino acids in the bloodstream [61,62]. Therefore, the reduction of these amino acids following DXT-M treatment reflects a restoration of hepatic aminotransferase function and ammonia detoxification capacity. Finally, the close clustering of complement C3 with traditional liver function tests (ALT, AST, ALP) and its significant correlation with specific metabolites reveals an intriguing interface between innate immunity and metabolism in this injury model. The complement system is a known mediator of inflammatory liver injury. The correlation pattern suggests that the metabolic stress signalled by perturbations in lipids and glutamate may be linked to complement activation [63,64]. DXT-M’s ability to concurrently normalise complement C3 levels and correct these metabolic disturbances points to its dual anti-inflammatory and metabolic-regulatory actions, which collectively contribute to its overall hepatoprotective efficacy.

The analysis revealed that in ANIT-induced acute liver injury models, there was a significant decrease in glucose levels, a marked increase in lipid levels, and the occurrence of recumbency and rapid weight loss. These findings indicate a disruption in energy and substance metabolism. Previous research has established that glucose is crucial to the body’s energy metabolism, as it produces pyruvate [65]. Pyruvate is then subjected to aerobic oxidation, resulting in the production of carbon dioxide and water, or anaerobic fermentation in yeast, leading to the formation of lactic acid or ethanol, thereby generating ATP to support the body’s essential functions [66]. The tricarboxylic acid (TCA) cycle, a crucial step in aerobic oxidation, is closely linked to the body’s energy metabolism [67,68,69]. In addition, in animals with liver injury, there was a marked reduction in the levels of citrate and α-ketoglutarate within the TCA cycle pathway compared to healthy controls. This diminution in TCA cycle intermediates resulted in impaired energy metabolism. Conversely, administration of DXT-M significantly increased the endogenous biomarkers of citric acid and α-ketoglutarate levels within the TCA cycle. Additionally, DXT-M effectively modulated the dysregulated glucose and lipid metabolism in liver-injured animals, thereby ameliorating their abnormal physiological indicators. Consequently, DXT-M is proposed as a potential therapeutic intervention for enhancing energy metabolism in liver-damaged animals by modulating the TCA cycle and regulating glucose metabolism.

The TCA cycle is a crucial intermediary pathway connecting various nutrients, including sugars, lipids, and proteins [70,71,72]. It facilitates the renewal and metabolism of amino acids through processes such as amino acid transamination, deamination, and α-ketoglutaric acid metabolism [73,74]. Following acute liver injury induced by ANIT, transaminases such as ALT and AST are released into the bloodstream, resulting in an abnormal elevation of serum ALT and AST levels. In the presence of aminotransferase, the TCA cycle intermediate α-ketoglutaric acid accepts amino groups to form glutamic acid, thereby expediting the depletion of α-ketoglutaric acid intermediates and diminishing energy metabolism within the TCA cycle. Furthermore, a reciprocal transformation and equilibrium dynamic exist between glutamate and glutamine, facilitated by the enzymatic activities of glutamine synthetase and glutaminase [75,76]. Glutamine serves as a detoxification agent for ammonia and plays a crucial role in the storage and transportation of ammonia [77,78,79]. In liver injury, hepatocytes sustain damage, disrupting the metabolism of glutamine and glutamate [75]. ANIT-induced liver injury leads to a substantial increase in glutamate levels and a significant decrease in glutamine levels. However, treatment with DXT-M effectively restores the balance of glutamate and glutamine metabolism, indicating its potential in liver detoxification through regulating glutamate-glutamine equilibrium. In instances of liver injury, aside from the previously mentioned impairments in glucose metabolism and the tricarboxylic acid (TCA) cycle, there is also a potential for disruptions in protein and lipid metabolism, as well as other substances [80,81,82].

The liver plays a crucial role in metabolism, detoxification, and immune regulation [83,84]. According to the Zangxiang theory in TCM, the liver aids in blood storage and relief functions [85]. The liver’s ability to store and circulate blood aids nutrient transformation and distribution in the body [86]. However, when pathogens invade, this function is disrupted, causing depression, impaired fluid and qi flow, reduced physical activity, and weakened nourishment [87]. Contemporary studies have demonstrated that inadequate warmth and nourishment provided by the body’s qi and blood can result in symptoms such as stagnation of qi, deficiency of qi, and stasis of blood, often accompanied by varying degrees of abnormal metabolism of substances and energy, primarily caused by disruptions in the TCA cycle [88,89,90]. The study found that animals with liver injury showed signs like recumbency and weight loss, along with abnormal liver function and energy metabolism issues related to the TCA cycle. These findings support the Zangxiang theory’s view of liver dysfunction. After administering DXT-M, these abnormalities improved, indicating that DXT-M has the potential to correct metabolic imbalances in liver injury by affecting TCA cycle biomarkers. The research highlights the metabolic profile of liver injury caused by ANIT and explains how DXT-M provides liver protection. In summary, the coordinated normalisation of this interconnected metabolite–clinical index network, as deciphered from Figure 6B, substantiates the multitarget and system-level therapeutic mechanism of DXT-M.

### 3.2. Bidirectional Regulation Network of DXT-M’s Hepatoprotection and Hepatotoxicity

Integrated metabolomics and network pharmacology revealed that DXT-M could modulate endogenous metabolites via specific targets, influencing hepatic energy metabolism and hepatoprotection. DXT-M components regulated ethanol and sugars through LDHA, impacting glycolysis and hepatic energy balance [91,92]. It could also modulate metabolites, such as phenylalanine, via targets (MPO, GSTM1, GSTP1, CYP450s, e.g., CYP3A4, CYP1A2), affecting tyrosine metabolism and linking to antioxidant/detoxification effects [93,94,95]. Furthermore, DXT-M components could influence glutamic acid, glycine, and 2-ketoglutaric acid through specific targets (GSTM1, GSTP1, NOS3), thereby regulating the urea cycle and arginine/proline/glutamic acid pathways, and maintaining a balance in nitrogen metabolism and supporting liver detoxification/repair [96,97,98]. DXT-M also modulated tryptophan metabolism via targets (GSTM1, GSTP1, CYP1A1, CYP1A2, CYP2B6, CYP3A4), affecting inflammatory factors [99,100]. Additionally, DXT-M and its components interact with bile acid receptors (BSEP, MRP2, and NSTP) and regulate conjugation molecules (glycine and taurine), thereby influencing bile acid biosynthesis and enterohepatic circulation, which contributes to hepatoprotection [41,43,101]. Key enzymes identified (CYP, GST, NOS3) significantly mediate DXT-M’s regulation of hepatic energy metabolism and oxidative stress through pathways including pyruvate-aspartate-glutamate metabolism, taurine metabolism, and the TCA cycle.

According to TCM principles, DXT, a prescription for damp-heat jaundice, exerts hepatoprotective effects when applied in conjunction with syndrome differentiation; however, its intrinsic properties can cause adverse effects if misused [30,102,103]. Network toxicology identified overlapping targets of hepatic drug-metabolising enzymes (CYP, GST) in DXT-M’s hepatoprotective (metabolomics-network pharmacology) and hepatotoxic mechanisms. Core pathway enrichment revealed 14 shared pathways (e.g., detoxification, energy metabolism, inflammation) and distinct hepatoprotection-specific pathways [104,105]. Crucially, the CYP450-GST (detoxification)-ROS (inflammation) axis (targets: CYP2B6, CYP1A1, CYP1A2, CYP3A4, GSTM1, GSTP1, IL1B, MMP2, TNF) represented the common intersection. CYP450 and GST facilitate xenobiotic detoxification via Phase I/II metabolism, but can also generate toxic metabolites that saturate GST capacity, leading to ROS accumulation, oxidative stress, inflammation (TNF-α, IL-1β), mitochondrial dysfunction, and disrupted TCA cycle energy metabolism, ultimately causing hepatotoxicity [106,107,108,109]. The GST/ROS balance determines the outcome of detoxification versus toxicity. DXT-M can ameliorate TCA cycle dysregulation (linked to hepatoprotection) and act via targets (e.g., NOS3, JUN, TP53) in specific pathways to promote vascular remodelling and mitochondrial function.

### 3.3. Translational Implications and Future Perspectives

The observed correlation between metabolic reprogramming patterns and pharmacodynamic improvements underscored the hepatoprotective efficacy of DXT-M in acute liver injury, suggesting its significant potential as a candidate for managing this condition. Integrated analyses of network pharmacology and toxicology revealed that key enzymes mediating the hepatoprotective effects of DXT-M, such as CYP450s and GSTs, may also serve as potential targets for its toxicity, indicating a dual-faceted mechanism inherent to its composition [109,110,111]. Future research should prioritise the experimental validation of this regulatory network using targeted approaches, such as siRNA or pharmacological inhibitors. Building upon this foundation, a promising future direction is the development of a more refined, multi-component formulation based on the representative bioactive compounds of DXT-M’s constituent herbs (e.g., anthraquinones, berberine, and geniposide). This strategy will help identify the core synergistic combination responsible for the observed efficacy and further elucidate the precise molecular mechanisms. Additionally, clinical translation will necessitate careful patient stratification and monitoring of metabolic enzyme activities to ensure safe application within a personalised medicine framework [112]. Given the identified overlap between efficacy and toxicity targets, the clinical translation of DXT-M as a multitarget therapeutic agent necessitates a personalised medicine approach [113]. Future clinical applications should involve careful patient stratification, and monitoring liver function and drug-metabolising enzyme activity (e.g., CYP3A4) would be crucial to mitigate potential herb–drug interactions or toxicity in susceptible individuals. Well-designed clinical trials are crucial for translating preclinical findings into safe and effective patient care.

Several limitations warrant consideration in interpreting these findings. The use of a single animal model (ANIT-induced acute liver injury) may not fully recapitulate the complexity of human liver diseases, and the absence of pharmacokinetic data for key active components limits understanding of their in vivo behaviour [114]. Most importantly, as these findings are derived solely from preclinical experiments, future research should incorporate clinical data through retrospective analyses of patient cases to validate the relevance of the identified metabolic profiles and molecular targets in human populations [115,116]. Such clinical correlation will be essential for translating these systemic insights into safe and effective therapeutic strategies.

## 4. Materials and Methods

### 4.1. Materials and Reagents

Rhei Radix Et Rhizoma (RRR; the dried root and rhizome of *Rheum palmatum* L.), Phellodendri Chinensis Cortex (PCC; the dried cortex of *Phellodendrn chinense* Schneid), Gardeniae Fructus (GF; the dried ripe fruit of *Gardenia jasminoides* Ellis.), Natrii Sulfas (NS; the kind of sulfate crystal mineral drug mainly containing Na_2_SO_4_·10H_2_O), were purchased from Shanxi Wanmin Pharmacy (Taiyuan, China). Aloe-emodin, rhein, emodin, chrysophanol, physcion, berberine, and geniposide were purchased from the National Institutes for Food and Drug Control (Beijing, China). α-naphthalene isothiocyanate (ANIT) was purchased from ALFA Aesar Chemical (Tianjin, China), and Ursodeoxycholic acid tablets were purchased from Shanghai Xinyi Pharmaceutical (Beijing, China). 3-Trimethylsilyl-propionate-d_4_ sodium (TPS) and deuteriumoxide (D_2_O) were purchased from Beijing Bailingwei Technology (Beijing, China). Glutamate pyruvate transaminase (ALT/GPT), glutamic oxalacetic transaminase (AST/GOT), alkaline phosphatase (ALP), total bilirubin (TBIL), indirect bilirubin (DBIL) and total bile acid (TBA) kits were purchased from Siemens Healthineers (Shanghai, China). Prealbumin (PA), Complement C3 (C3) and C4 kits were purchased from Ningbo Meikang Bioengineering (Ningbo, China). Interleukin-2 (IL-2), Interleukin-6 (IL-6) and tumour necrosis factor α (TNF-α) kits were purchased from Shanghai Bioengineering (Shanghai, China).

### 4.2. Herbal Refined Extracts Preparation

DXT was prepared by boiling a mixture of RRR (12 g), PCC (12 g), GF (9 g), and NS (12 g) according to the original prescription ratio using 10 volumes of water per extraction for two cycles, with the combined filtrate concentrated to 0.5 g crude drug per mL [29]; DXT-M was formulated from refined extracts of RRR, PCC, and GF combined with native NS in the optimised ratio of RRR refined extract 0.8478 g, PCC refined extract 0.3150 g, GF refined extract 0.9990 g, and NS 4 g [41]. The individual refined extracts were prepared as follows: RRR (1000 g) was extracted three times with water (1:10, *w*/*v*, 30 min each), and the combined filtrates were concentrated to 0.75 g/mL (equivalent to 0.75 g crude drug per mL) before purification on an AB-8 macroporous resin column (loaded at 0.94 g crude drug per mL resin), where the column was washed with water until the effluent became clear and then eluted with 95% ethanol until the eluate was colorless, with the ethanol eluate concentrated under reduced pressure to a thick extract and dried at low temperature to yield 94.2 g of refined extract; PCC (1000 g) was refluxed three times with 75% ethanol (1:10, *w*/*v*, 1.5 h each), and after ethanol recovery, the combined filtrate was concentrated to 0.5 g/mL (equivalent to 0.5 g crude drug per mL) and processed on an AB-8 resin column (loaded at 1 g crude drug per mL resin) with water wash until clear effluent followed by elution with 50% ethanol until the resin bed became colorless, with the ethanol eluate concentrated under reduced pressure to a thick extract and dried at low temperature to yield 52.5 g of refined extract; GF (1000 g) was decocted twice with water (1:12, *w*/*v*, 1 h each), and the combined filtrate was concentrated to 0.5 g/mL (equivalent to 0.5 g crude drug per mL) before separation on an HPD-100 resin column (loaded at 1.5 g crude drug per mL resin) using water wash until clear effluent and subsequent elution with 75% ethanol until the eluate became colorless, with the ethanol eluate concentrated under reduced pressure to a thick extract and dried at low temperature to yield 74.0 g of refined extract [45,47].

### 4.3. Qualitative Analysis of RRR, PCC and GF

0.1 g of RRR, PCC, and GF dried herbal extracts were each dissolved in methanol in 10 mL flasks and then filtered through a 0.22 μm membrane. 0.76 mg aloe-emodin, 0.41 mg rhein, 0.92 mg emodin, 1.17 mg physcion, and 0.51 mg chrysophanol were dissolved separately in methanol to a total volume of 10 mL. Mix 2 mL of each to form a mixed reference solution of anthraquinones. Reference solutions were also prepared by dissolving 1.00 mg of berberine and 0.30 mg of geniposide in methanol to a final volume of 10 mL, separately. The chemical constituents of RRR, PCC, GF extracts and their reference substances were analysed using Agilent 1260 HPLC (Santa Clara, CA, USA). The YMC-Pack ODS-AD Column (Kyoto, Japan) was used for RRR and GF, while a Phenomenex Gemini-NX Column (Torrance, CA, USA) was used for PCC. Elution details and detection wavelengths are in Table 1. NS, primarily sodium sulfate decahydrate (Na_2_SO_4_·10H_2_O), was not further analysed due to its known composition [117,118].

### 4.4. Animals Feeding

Equal numbers of male and female Wistar rats (200 ± 20 g) were provided by the Experimental Animal Center, Academy of Military Medical Sciences (Beijing, China, SCXK (Army) 2012-0004). They were fed standard food and water for 7 days under a 12 h light/dark cycle at 20 ± 2 °C and 40 ± 5% humidity to acclimate. Throughout the study, every effort was made to minimise animal suffering and stress. All procedures were performed by personnel trained in rodent handling, using gentle restraint to minimise the duration of any discomfort. The housing conditions were strictly maintained to ensure animal well-being. Experiments were conducted in accordance with the guidelines of the Animal Ethics Committee of Shanxi University of Chinese Medicine, ensuring minimal animal suffering (Approval number: AWE2025093635).

### 4.5. Animal Grouping, Modelling and Administration Procedure

**Figure 11:**
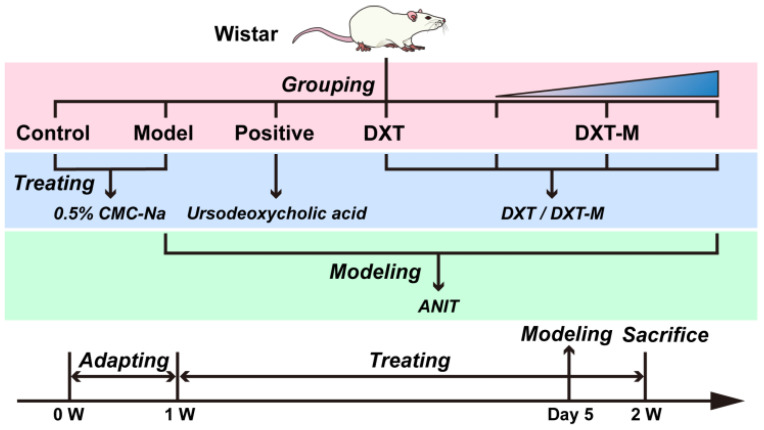
Schematic illustration of the animal experimental procedure and timeline.

Wistar rats (*n* = 6 per group, balanced by sex) were randomly divided into seven groups: control, model, positive (ursodeoxycholic acid), DXT, low, medium, and high doses of DXT-M (5.4, 10, and 20 times the human equivalent dose). After a 7-day adaptation period, the control and model groups received a daily 0.5% sodium carboxymethylcellulose (CMCNa) solution (1 mL/100 g) for 7 days. Other groups received an equal volume of drug solution with 0.5% CMCNa solution. On day five, 2 h post-administration, control rats received olive oil (1 mL/100 g), while others got ANIT (60 mg/kg in olive oil) to induce acute jaundiced liver injury. Daily body weight and biological signs were monitored. The flowchart of the animal experiment is depicted in Figure 11.

### 4.6. Biological Samples Collection and Preparation

After administration on day 6, animals from the control, model, and medium dose DXT-M groups were placed in metabolic cages to collect urine over a 24 h period. The urine was centrifuged at 3000 rpm for 15 min, and the supernatant was stored at −80 °C. On day 7, two hours post-administration, animals were deeply anesthetised with isoflurane to ensure complete loss of consciousness before blood collection and subsequent euthanasia. Euthanasia was confirmed by the absence of pedal reflex. The blood was left at room temperature for 30 min, then centrifuged at 3000 rpm for 15 min at 4 °C to obtain serum, which was stored at −80 °C.

### 4.7. Biochemical and Pathological Test

Serum biochemical indicators were evaluated using kits, while liver function and metabolism markers (ALT, AST, ALP, TBIL, DBIL, TBA) were assessed through chemical colourimetric analysis. PA and Complement levels (C3, C4) were measured via immunoturbidimetry, and TNF-α, IL-2, and IL-6 levels were determined using ELISA. After humane euthanasia, rat livers were fixed in formalin, paraffin-embedded, *H&E*-stained, and examined under a 100× magnification Olympus DP80 Microscope (Tokyo, Japan).

### 4.8. Metabolomics Study Based on ^1^H NMR Analysis

The serum and urine samples were thawed on ice for NMR analysis. 350 μL of serum was mixed with 250 μL of D_2_O, vortexed, and then centrifuged. The supernatant was subsequently placed in an NMR tube. 500 μL of urine was combined with 200 μL of PBS buffer, vortexed, and then centrifuged. The supernatant was subsequently transferred to an NMR tube. ^1^H-NMR spectra were obtained using a 600 MHz Bruker spectrometer with standard parameters. Data were processed with MestReNova, and the spectra were segmented, normalised, and analysed using principal component analysis (PCA) and orthogonal partial least squares discriminant analysis (OPLS-DA) in SIMCA-P. Metabolites with variable importance plot (*VIP*) > 1 and *p* < 0.05 were deemed significant, and hierarchical clustering visualised metabolite patterns.

### 4.9. Integrated Analysis Workflow

#### 4.9.1. Procedure for Target Screening

Compounds from RRR, PCC and GF were selected from the TCMSP Database based on oral bioavailability (OB) ≥ 30% and drug-likeness (DL) ≥ 0.18. Protein targets were converted to gene names via Uniprot to form a DXT-M target database. Search for hepatitis-related targets using “hepatitis” in OMIM, GeneCards, TTD, and DisGeNET, and include clinical drug targets from DRUGBANK. Combine and remove duplicates to create a comprehensive database of hepatitis targets. Use Venny 2.1.0 to visualise overlaps between DXT-M and hepatitis targets post-deduplication.

#### 4.9.2. Construction of the Integrated Network

Intersection targets from Section 4.9.1 were imported into Cytoscape v3.8.2 using the Metscape plug-in to explore potential targets in the metabolite–reaction–enzyme–gene network. Targets regulated in this network, as well as differential metabolites from Section 4.8, were also imported into Metscape. Consequently, compound–reaction–enzyme–gene networks linked to metabolite enrichment pathways were constructed.

#### 4.9.3. Target Screening, Target Interaction, and Enrichment Analysis

Potential targets were added to the STRING Database to create a PPI network, analysed using Cytoscape v3.8.2. The MCODE plug-in identified the core subnetwork, highlighting key targets. These targets, along with those from item 2.9.2, underwent GO and KEGG enrichment analysis via the DAVID Database with *p* < 0.05. For further analysis, GO entries and KEGG pathways were selected based on *p* values. Results were visualised on WeiShengXin, and Sankey diagrams were generated using CNSknowall to connect core targets with GO items or KEGG pathways.

### 4.10. Network Toxicology Workflow

The Comparative Toxicogenomics Database was used to filter genes linked to drug-induced liver injury to create a hepatotoxicity target database. Venny 2.1.0 identified overlapping targets with the DXT-M component database, forming the DXT-M hepatotoxicity target database. STRING analysed these targets’ PPI, and Cytoscape v3.8.2 with MCODE identified core subnetworks. Key targets were identified through network topology and analysed for GO and KEGG pathways using the DAVID Database.

### 4.11. Target–Component Fitting Procedures

3D protein structures were sourced from the Protein Data Bank and UniProt, then refined with MOE software v2018.10. Key constituents of DXT-M were optimised via energy minimisation. Potential protein binding sites were identified using endogenous ligands or site finders. Rigid docking of DXT-M’s chemical constituents with target amino acids was facilitated by the London dG and GBVI/WSA dG scoring methods. Ligand–receptor affinities were evaluated using fitting scores, protein–ligand interaction fingerprints (PLIF), and 3D interaction analyses.

### 4.12. Bidirectional Regulatory Network of DXT-M in Hepatoprotection and Hepatotoxicity

The core hepatoprotective and hepatotoxicity targets of DXT-M were merged, deduplicated, and analysed for KEGG pathway enrichment using the DAVID Database. The bidirectional regulatory network of DXT-M’s liver effects was visualised as a Sankey diagram on the CNSknowall platform.

### 4.13. Statistical Analysis

Statistical analyses were conducted using GraphPad Prism (v10.1.2), R (v4.2.1) and DPS software (21.05) [119]. Data normality was checked with the Shapiro–Wilk test. For normal data, Repeated measures (RM) two-way ANOVA with the Geisser–Greenhouse correction and Dunnett’s post hoc test were used to analyse body weight and longitudinal data, while one-way ANOVA with Dunnett’s post hoc test was used to compare serum markers, cytokines, and metabolites. Results were shown as mean ± standard deviation (SD). Hierarchical clustering heatmaps used Euclidean distance and complete linkage. Spearman’s rank correlation assessed metabolite and clinical index correlations.

## 5. Conclusions

This study systematically elucidated the multidimensional “phenotype–metabolism–target” mechanism of DXT-M in combating liver injury. This was achieved by integrating animal experiments, untargeted metabolomics, and network pharmacology–toxicology analysis. The findings revealed that DXT-M significantly ameliorated ANIT-induced acute liver injury phenotypes, normalised liver function parameters such as transaminase, bilirubin, and bile acid levels, and restored the status of the complement system, while also repairing hepatic pathological damage. DXT-M achieved this by modulating a network of metabolites (including citrate, taurine, glutamate, alanine, and others), which interact with targets of hepatic drug-metabolising enzymes, NOS3, and LDHA, thereby correcting endogenous metabolic disorders through intrinsic target molecules. Notably, CYP/GST acted as a bidirectional regulatory switch between hepatoprotection (detoxification) and ROS (hepatotoxicity), while NOS3/TP53 function as synergistic hepatoprotective targets, as confirmed by molecular docking studies showing high residual affinity. The bidirectional regulation mechanism of DXT-M’s hepatoprotective and hepatotoxic effects requires further elucidation. Future studies should focus on further validating this regulatory network, elucidating the complete material basis, and conducting clinical retrospective analyses to translate these findings into safe and effective therapies.

## Figures and Tables

**Table 1:** Elutions and detection wavelengths for DXT and DXT-M compositions.

Time/Min	A (Acetonitrile + 0.0375% TFA)	B (Water + 0.0375% TFA)	Detective Wavelengths	Compositions
0	0	100%	380 nm	RRR
15	15%	85%		
35	30%	70%		
40	35%	65%		
50	40%	60%		
55	60%	40%		
60	100%	0		
65	100%	0		
0	0	100%	345 nm	PCC
7	20%	80%		
12	25%	75%		
18	80%	20%		
25	80%	20%		
0	0	100%	238 nm	GF
30	25%	75%		
50	40%	60%		

## Data Availability

Data presented in this study is contained within the article. Further inquiries can be directed to the corresponding authors.

## Supplementary Materials

The following supporting information can be downloaded at: https://www.mdpi.com/article/10.3390/ph18101534/s1, Figure S1: HPLC chromatograms of DXT and DXT-M compositions. Figure S2: Representative ^1^H-NMR spectra of control, model, and DXT-M treated groups. Figure S3: Protein–ligand interaction fingerprints (PLIF) of DXT-M target binding modes. Table S1: Gradient elution conditions and detection methods for DXT&DXT-M compositions. Table S2: Differential metabolites identified in serum and urine metabolomics analysis. Table S3: Topological analysis of core target proteins in network pharmacology. Table S4: Topological analysis of core target proteins in network toxicology. Table S5: Fitness scores of DXT-M representative components with core targets.

## Abbreviations

DXT	Dahuang Xiaoshi Tang
RRR	Rhei Radix Et Rhizoma
PCC	Phellodendri Chinensis Cortex
GF	Gardeniae Fructus
NS	Natrii Sulfas
ANIT	α-Naphthalene isothiocyanate
ALT/GPT	Glutamate pyruvate transaminase
AST/GOT	Glutamic oxalacetic transaminase
ALP	Alkaline phosphatase
TBIL	Total bilirubin
DBIL	Indirect bilirubin
TBA	Total bile acid
C3	Complement 3
C4	Complement 4
PA	Prealbumin
IL-2	Interleukin-2
IL-6	Interleukin-6
TNF-α	Tumour necrosis factor α
TCM	Traditional Chinese medicine
HPLC	High Performance Liquid Chromatography
HE	Hematoxylin-eosin
PCA	Principal component analysis
OPLS-DA	Orthogonal partial least squares-discriminant analysis
*VIP*	Variable importance plot
PPI	Protein–protein interaction
GO	Gene Ontology
BP	Biological Process
CC	Cellular Component
MF	Molecular Function
TCA	Tricarboxylic acid cycle
GSH	Glutathione
CYP450	Cytochrome P450
GST	Glutathione S-transferase
