# Supplementary material for: The Hepatoprotective Properties of the Revised Formulation of Dahuang Xiaoshi Tang, an Ancient Chinese Herbal Decoction, Are Probed by Integrated Metabolomics and Network Pharmacology
Source: Pharmaceuticals (Basel). 2025 Oct 13;18(10):1534. doi: 10.3390/ph18101534 (PMC12567495; doi:10.3390/ph18101534)
Supplement: Supplementary file 1 [file pharmaceuticals-18-01534-s001.zip › pharmaceuticals-3885324-supplementary.pdf]

**Supplementary Table**

**Table S1.** Composition and key phytochemical constituents of DXT.

| Composition | Main Constituent | Molecular Formula | Content | Activity | Reference |
|---|---|---|---|---|---|
| Rhei Radix Et Rhizoma (RRR) | Aloe-emodin | $C_{15}H_{10}O_5$ | 0.17 - 1.58 mg/g | Lipid and cholesterol regulation; Anti-hepatic fibrosis; Stool and intestinal barrier protection; Anti-inflammatory and antioxidant | [31-34] |
| | Rhein | $C_{15}H_8O_6$ | 0.22 - 4.57 mg/g | | |
| | Emodin | $C_{15}H_{10}O_5$ | 0.45 - 5.43 mg/g | | |
| | Chrysophanol | $C_{15}H_{10}O_4$ | 0.22 - 6.85 mg/g | | |
| | Physcion | $C_{16}H_{12}O_5$ | 0.22 - 2.76 mg/g | | |
| Phellodendri Chinensis Cortex (PCC) | Berberine | $C_{20}H_{18}NO_4$ | 2.13 %- 7.68 % | Anti-hepatic fibrosis; Lipid-regulation; Anti-inflammatory and antioxidant | [35,36] |
| Gardeniae Fructus (GF) | Geniposide | $C_{17}H_{24}O_{10}$ | 1.80 %- 2.04 % | Hepatoprotection; Cholagogue; Anti-inflammatory and antioxidant; Neuroprotective | [37,38] |
| Natrii Sulfas (NS) | Sodium sulfate decahydrate | $Na_2SO_4 \cdot 10H_2O$ | ＞ 99 % ($Na_2SO_4$) | Stimulates gastrointestinal motility; Promotes water discharge; Lowers cholesterol; Anti-inflammatory & detumescent | [117,118] |

Note: The anthraquinone components in RRR were expressed as free anthraquinone.

**Table S2.** Differential metabolites identified in serum and urine metabolomics analysis.

| Source | No. | HMDB ID | KEGG ID | Metabolites | $\delta_H$ |
|---|---|---|---|---|---|
| Serum | 1 | - | - | Lipid | **0.88 (m), 1.28 (m), 2.76 (m), 2.80 (m)** |
| Serum | 2 | HMDB0000108 | C00469 | Ethanol | **1.20 (t)** |
| Serum | 3 | HMDB0000167 | C00188 | Threonine | **1. 32 (d)** |
| Serum | 4 | HMDB0000148 | C00025 | Glutamic acid | **2.04 (td)** |
| Serum | 5 | HMDB0000251 | C00245 | Taurine | **3.24 (t), 3.44 (t)** |
| Serum | 6 | HMDB0000122 | C00221 | Glucose | **3.48 (dd)** |
| Serum | 7 | HMDB0000211 | C00137 | myo-Inositol | **3.56 (dd)** |
| Serum | 8 | HMDB0000641 | C00064 | Glutamine | **3.80 (t)** |
| Urine | 1 | HMDB0000161 | C00041 | Alanine | **1.48 (d)** |
| Urine | 2 | HMDB0000510 | C00956 | Aminoadipic acid | **1.60-1.88 (m)** |
| Urine | 3 | HMDB0000182 | C00047 | Lysine | **1.76 (tt), 1.92 (dtd)** |
| Urine | 4 | HMDB0000719 | C00263 | Homoserine | **3.78 (m), 3.84 (dd)** |
| Urine | 5 | HMDB0000208 | C00026 | Oxoglutaric acid | **2.45 (m), 3.02 (m)** |
| Urine | 6 | HMDB0000094 | C00158 | Citric acid | **2.52 (d), 2.69 (d)** |
| Urine | 7 | HMDB0000164 | C00218 | Methylamine | **2.61 (s)** |
| Urine | 8 | HMDB0000159 | C00079 | Phenylalanine | **3.14 (m), 3.28 (m)** |
| Urine | 9 | HMDB0000123 | C00037 | Glycine | **3.57 (s)** |
| Urine | 10 | HMDB0000821 | C05598 | Phenylacetylglycine | **3.68 (s), 3.76 (d)** |
| Urine | 11 | HMDB0000714 | C01586 | Hippuric acid | **3.97 (d), 7.56 (m), 7.64 (tt), 7.84 (dd)** |

Note: Metabolites in bold indicate those with corresponding peaks labeled in the $^1$H-NMR spectrum **(Supplenmentary Figure 4)**.

**Table S3.** Topological analysis of core target proteins in network pharmacology.

| No. | Target | Degree | Betweenness centrality | Closeness centrality | Clustering coefficient |
|---|---|---|---|---|---|
| 1 | TP53 | 120 | 0.043539055 | 0.619047619 | 0.389830508 |
| 2 | EGFR | 120 | 0.085238906 | 0.639344262 | 0.360451977 |
| 3 | TNF | 112 | 0.050448278 | 0.615789474 | 0.398701299 |
| 4 | IL1B | 106 | 0.038622453 | 0.612565445 | 0.425979681 |
| 5 | JUN | 104 | 0.030743312 | 0.603092784 | 0.45173454 |
| 6 | PTGS2 | 76 | 0.041221276 | 0.570731707 | 0.520625889 |
| 7 | MMP2 | 54 | 0.013814016 | 0.52 | 0.464387464 |
| 8 | HRAS | 44 | 0.003805762 | 0.49787234 | 0.601731602 |
| 9 | *MPO | 26 | 0.034212562 | 0.441509434 | 0.653846154 |
| 10 | *CYP1A1 | 22 | 0.016611441 | 0.409090909 | 0.381818182 |
| 11 | *GSTP1 | 22 | 0.028220171 | 0.45 | 0.290909091 |
| 12 | *NOS3 | 20 | 0.001072795 | 0.464285714 | 0.577777778 |
| 13 | *CYP2B6 | 18 | 0.024423545 | 0.402061856 | 0.5 |
| 14 | *CYP3A4 | 18 | 0.004961561 | 0.346153846 | 0.527777778 |
| 15 | *CYP1A2 | 16 | 0.001702408 | 0.340116279 | 0.607142857 |
| 16 | CCNA2 | 16 | 1.24E-04 | 0.414893617 | 0.821428571 |
| 17 | LGALS3 | 16 | 0.017130581 | 0.458823529 | 0.642857143 |
| 18 | *GSTM1 | 12 | 0.004059452 | 0.362229102 | 0.666666667 |
| 19 | *LDHA | 4 | 0 | 0.376205788 | 1 |

Note: Target: Gene symbols are shown. Targets marked with asterisks (*) were identified through integrated network pharmacology and metabolomics analysis.

**Table S4.** Topological analysis of core target proteins in network toxicology.

| No. | Target | Degree | Betweenness centrality | Closeness centrality | Clustering coefficient |
|---|---|---|---|---|---|
| 1 | TNF | 62 | 0.147834014 | 0.79245283 | 0.374193548 |
| 2 | IL1B | 54 | 0.117949124 | 0.736842105 | 0.404558405 |
| 3 | CCL2 | 44 | 0.04942938 | 0.666666667 | 0.510822511 |
| 4 | MMP9 | 44 | 0.037521543 | 0.646153846 | 0.506493506 |
| 5 | HMOX1 | 42 | 0.056936515 | 0.666666667 | 0.495238095 |
| 6 | PPARA | 40 | 0.045831486 | 0.65625 | 0.526315789 |
| 7 | IFNG | 38 | 0.015983398 | 0.6 | 0.602339181 |
| 8 | *CYP3A4 | 38 | 0.053729046 | 0.626865672 | 0.497076023 |
| 9 | *CYP1A1 | 36 | 0.040921222 | 0.617647059 | 0.562091503 |
| 10 | MMP2 | 34 | 0.023304205 | 0.6 | 0.617647059 |
| 11 | *CYP1A2 | 32 | 0.018382553 | 0.552631579 | 0.583333333 |
| 12 | *CYP2B6 | 32 | 0.017770295 | 0.545454545 | 0.566666667 |
| 13 | *GSTP1 | 26 | 0.015400272 | 0.567567568 | 0.641025641 |
| 14 | *GSTM1 | 26 | 0.014548211 | 0.567567568 | 0.641025641 |

Note: Target: Gene symbols are shown. Targets marked with asterisks (*) were identified through integrated network pharmacology and metabolomics analysis.

**Table S5.** Fitness scores of DXT-M representative components with core targets.

| | | **Compounds scores** | | | | | | | | | | | |
|---|---|---|---|---|---|---|---|---|---|---|---|---|---|
| | | **aloe-emodin** | **rhein** | **emodin** | **chrysophanic acid** | **geniposide** | **crocetin** | ***chromone** | **berberine** | **palmatine** | **berberrubine** | **quercetin** | **β-sitosterol** |
| | **EGFR** | -5.86±0.29 | -5.75±0.32 | -5.52±0.29 | -5.4±0.31 | -7.06±0.23 | -6.55±0.06 | -6.58±0.29 | -6.32±0.27 | -6.66±0.25 | -5.98±0.29 | -5.8±0.28 | -6.57±0.35 |
| | **TP53** | -4.95±0.18 | -5.09±0.15 | -4.94±0.19 | -4.83±0.26 | -5.76±0.18 | -5.54±0.18 | -5.74±0.29 | -5.41±0.34 | -5.83±0.34 | -5.34±0.36 | -4.97±0.15 | -5.36±0.17 |
| | **TNF** | -5.74±0.2 | -5.86±0.17 | -5.63±0.16 | -5.55±0.2 | -7.51±0.41 | -7.26±0.12 | -7.73±0.43 | -6.75±0.44 | -6.84±0.29 | -6.48±0.45 | -6.95±0.19 | -6.92±0.66 |
| | **IL1B** | -4.72±0.17 | -4.7±0.13 | -4.63±0.21 | -4.64±0.19 | -6.18±0.18 | -5.75±0.56 | -5.69±0.2 | -5.04±0.35 | -5.35±0.33 | -5.04±0.37 | -5.2±0.24 | -5.96±0.28 |
| | **JUN** | -5.87±0.32 | -6.03±0.27 | -5.72±0.33 | -5.66±0.33 | -7.28±0.28 | -6.44±0.25 | -6.95±0.29 | -6.42±0.29 | -6.74±0.18 | -6.06±0.26 | -6.14±0.31 | -7.06±0.22 |
| | **LGALS3** | -4.94±0.11 | -4.99±0.14 | -4.87±0.1 | -4.8±0.09 | -5.86±0.13 | -5.99±0.11 | -5.79±0.18 | -5.34±0.17 | -5.5±0.16 | -5.02±0.13 | -5.06±0.12 | -5.53±0.21 |
| | **MMP2** | -5.49±0.24 | -5.63±0.29 | -5.28±0.32 | -5.17±0.38 | -6.5±0.41 | -7.57±0.15 | -6.82±0.27 | -6.35±0.21 | -6.76±0.19 | -5.97±0.41 | -6.23±0.14 | -6.67±0.24 |
| | **CCNA2** | -6.07±0.13 | -6.08±0.18 | -5.85±0.22 | -5.75±0.2 | -7.45±0.26 | -6.41±0.29 | -6.98±0.12 | -6.58±0.22 | -7.15±0.29 | -6.42±0.23 | -6.29±0.23 | -6.52±0.48 |
| | **PTGS2** | -6.35±0.33 | -5.75±1.6 | -4.8±1.88 | -5.29±1.63 | -5.42±0.48 | -7.03±0.22 | -6.26±0.34 | -3.56±1.34 | -4.57±0.82 | -3.58±1.38 | -5.76±0.66 | 0.65±1.77 |
| | **LDHA** | -5.27±0.15 | -5.3±0.2 | -5.17±0.17 | -5.05±0.17 | -6.8±0.22 | -6.34±0.31 | -6.42±0.1 | -5.82±0.06 | -6.29±0.11 | -5.72±0.14 | -5.53±0.14 | -7.09±0.19 |
| | **GSTM1** | -4.99±0.12 | -5.17±0.19 | -5.07±0.2 | -4.9±0.24 | -6.05±0.17 | -5.33±0.12 | -5.83±0.22 | -5.34±0.18 | -5.57±0.2 | -5.13±0.15 | -5.2±0.15 | -6±0.24 |
| **Targets** | **CYP1A1** | -7.71±0.38 | -7.69±0.5 | -7.2±0.75 | -7.2±0.73 | -8.8±0.22 | -9.4±0.14 | -9.18±0.34 | -8.37±0.61 | -9.02±0.5 | -7.95±0.63 | -7.92±0.3 | -6.55±0.8 |
| | **CYP1A2** | -7.52±0.2 | -7.33±0.31 | -7.1±0.45 | -7.15±0.49 | -8.74±0.28 | -9.44±0.12 | -8.68±0.19 | -8.09±0.67 | -8.51±0.84 | -7.77±0.54 | -7.34±0.27 | -5.96±0.57 |
| | **CYP3A4** | -5.59±0.18 | -5.62±0.28 | -5.35±0.21 | -5.27±0.13 | -7.01±0.19 | -6.88±0.16 | -7.08±0.14 | -6.45±0.2 | -6.77±0.21 | -6.27±0.22 | -6.04±0.13 | -7.97±0.18 |
| | **NOS3** | -5.95±0.09 | -6.1±0.15 | -5.86±0.11 | -5.75±0.14 | -7.06±0.18 | -7.08±0.38 | -7.26±0.34 | -6.88±0.37 | -7.37±0.37 | -6.58±0.34 | -6.19±0.19 | -6.98±0.23 |
| | **MPO** | -6.38±0.25 | -6.4±0.16 | -6.18±0.29 | -6.09±0.3 | -7.71±0.18 | -7.74±0.17 | -7.85±0.26 | -7.29±0.18 | -7.69±0.19 | -7.07±0.31 | -6.71±0.16 | -7.3±0.33 |
| | **MMP9** | -5.96±0.22 | -5.89±0.29 | -5.6±0.42 | -5.62±0.35 | -6.7±0.28 | -7.45±0.11 | -7.14±0.22 | -6.22±0.45 | -6.89±0.4 | -5.71±0.77 | -6.78±0.24 | -6.67±0.36 |
| | **HMOX1** | -5.33±0.15 | -5.43±0.13 | -5.29±0.15 | -5.18±0.16 | -6.76±0.2 | -6.39±0.21 | -6.66±0.18 | -5.93±0.17 | -6.23±0.17 | -5.81±0.18 | -5.48±0.15 | -6.53±0.26 |
| | **PPARA** | -5.8±0.3 | -5.83±0.36 | -5.37±0.65 | -5.37±0.54 | -7.01±0.27 | -7.8±0.22 | -7.14±0.31 | -6.26±0.47 | -7.19±0.34 | -5.62±0.8 | -5.91±0.3 | -4.33±0.74 |
| | **HRAS** | -5.12±0.13 | -5.13±0.14 | -4.98±0.25 | -4.87±0.18 | -6.18±0.23 | -5.93±0.25 | -6.32±0.38 | -5.46±0.31 | -5.82±0.31 | -5.3±0.26 | -5.33±0.15 | -6.11±0.24 |
| | **GSTP1** | -4.91±0.13 | -4.94±0.13 | -4.75±0.22 | -4.73±0.21 | -6.1±0.2 | -6.19±0.16 | -6.05±0.14 | -5.53±0.18 | -5.72±0.14 | -5.32±0.19 | -5.11±0.18 | -5.96±0.16 |
| | **CCL2** | -4.68±0.1 | -4.76±0.09 | -4.63±0.1 | -4.56±0.12 | -5.39±0.21 | -5.62±0.16 | -5.61±0.19 | -5.11±0.17 | -5.31±0.17 | -5.01±0.15 | -4.85±0.14 | -5.5±0.17 |
| | **IFNG** | -4.78±0.26 | -4.79±0.19 | -4.74±0.25 | -4.72±0.23 | -5.75±0.12 | -5.74±0.1 | -5.72±0.15 | -5.4±0.19 | -5.71±0.14 | -5.2±0.21 | -5.01±0.17 | -6.34±0.25 |

Note: *Chromone refers to 5-Hydroxy-7-methoxy-2-(3,4,5-trimethoxyphenyl)chromone. Scores are presented as mean ± SD of 20 independent docking runs.

**Supplementary Figure**

**Figure S1. HPLC chromatograms and main chemical components of DXT and DXT-M compositions.**

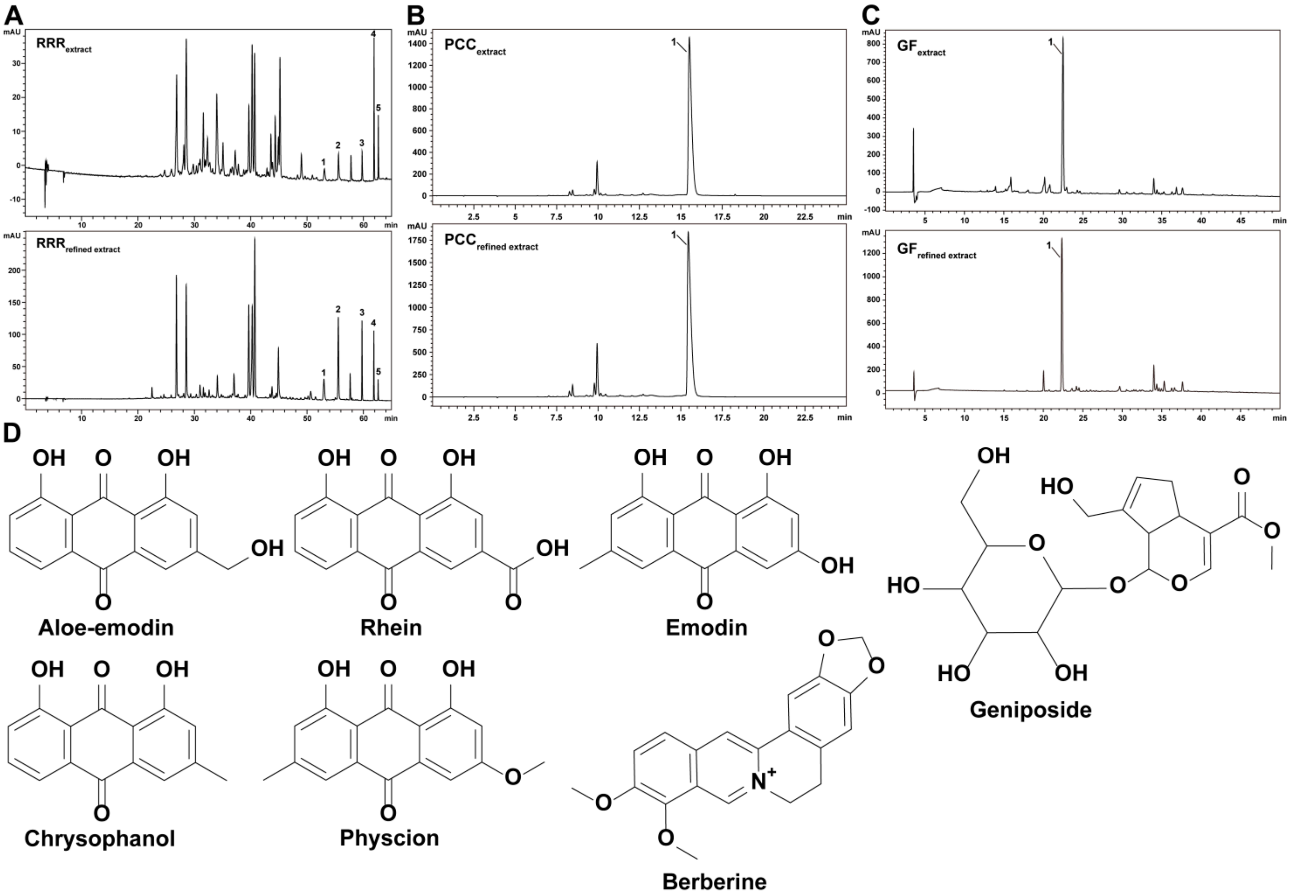


Note: **(A)** HPLC chromatograms of RRR extract before and after purification. Peaks are labeled as: 1-aloe-emodin ($t_R$ = 53.738 min), 2-rhein ($t_R$ = 55.962 min), 3-emodin ($t_R$ = 59.914 min), 4-chrysophanol ($t_R$ = 61.989 min), 5-physcion ($t_R$ = 62.756 min). **(B)** HPLC chromatograms of PCC extract before and after purification. Peaks are labeled as: 1-berberine ($t_R$ = 15.709 min). **(C)** HPLC chromatograms of GF extract before and after purification. Peaks are labeled as: 1-geniposide ($t_R$ = 22.516 min), 2-crocin ($t_R$ = 3.746 min). **(D)** The structures of representative phytochemicals in RRR, PCC and GF.

**Figure S2. Representative ¹H-NMR spectra of control, model, and DXT-M treated groups.**

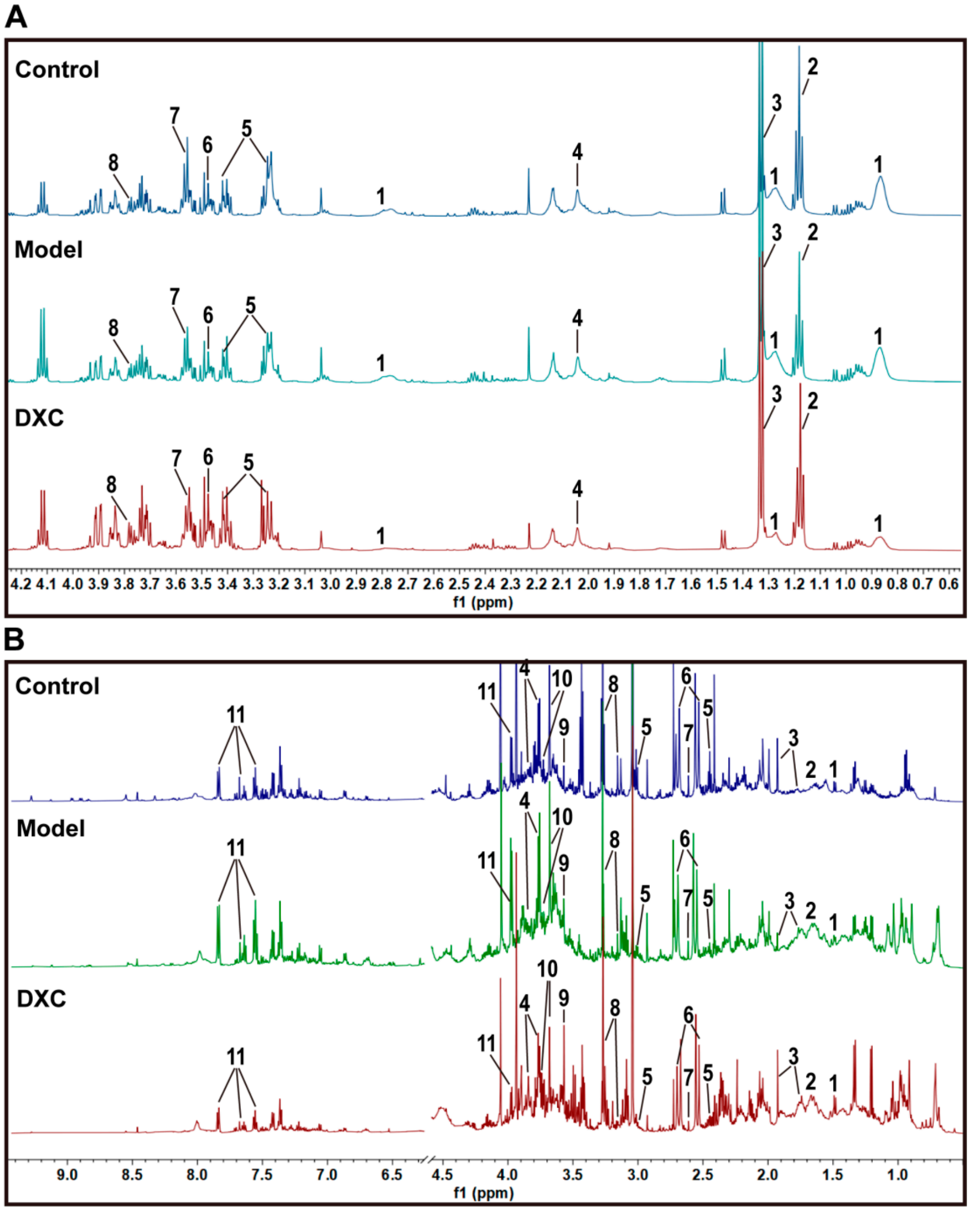


**(A)** Serum samples; **(B)** Urine samples. Numbers indicate annotated endogenous metabolite peaks identified by Chenomx NMR Suite 9.0, with corresponding metabolites listed in **Supplementary Table S2** (matched by numbering).

**Figure S3. Protein-ligand interaction fingerprints (PLIF) of DXT-M target binding modes.**

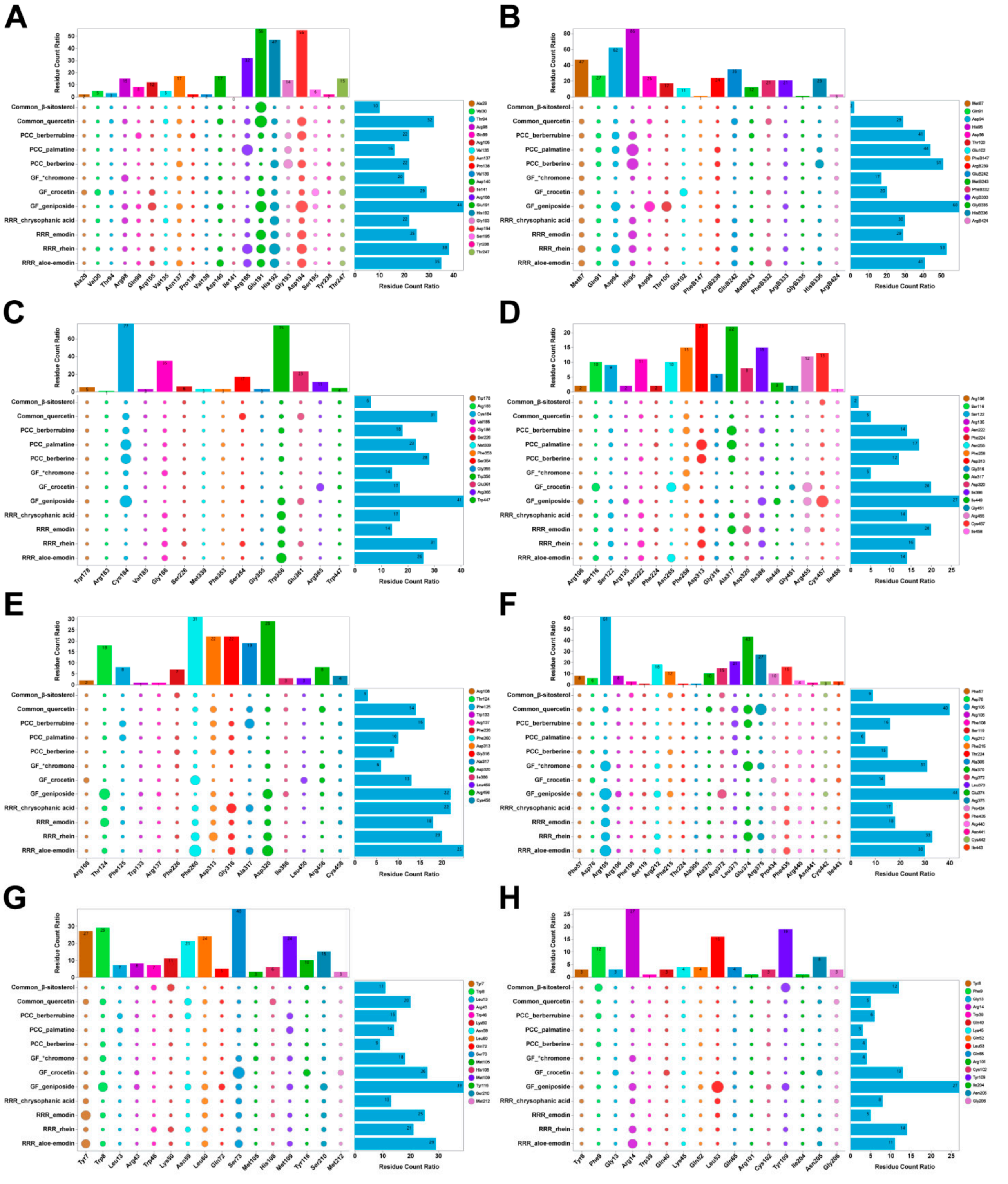


**(A-H)** Quantitative PLIF profiles showing residue-specific interactions between DXT-M compounds and eight targets: LDHA, MPO, NOS3, CYP1A1, CYP1A2, CYP3A4, GSTM1, and GSTP1. Bubble size corresponds to binding affinity, color coding indicates residue types, and column height represents cumulative residue affinity of corresponding row or column. *Chromone refers to 5-Hydroxy-7-methoxy-2-(3,4,5-trimethoxyphenyl) chromone.
